# A genome survey of *Moniliophthora perniciosa *gives new insights into Witches' Broom Disease of cacao

**DOI:** 10.1186/1471-2164-9-548

**Published:** 2008-11-18

**Authors:** Jorge MC Mondego, Marcelo F Carazzolle, Gustavo GL Costa, Eduardo F Formighieri, Lucas P Parizzi, Johana Rincones, Carolina Cotomacci, Dirce M Carraro, Anderson F Cunha, Helaine Carrer, Ramon O Vidal, Raíssa C Estrela, Odalys García, Daniela PT Thomazella, Bruno V de Oliveira, Acássia BL Pires, Maria Carolina S Rio, Marcos Renato R Araújo, Marcos H de Moraes, Luis AB Castro, Karina P Gramacho, Marilda S Gonçalves, José P Moura Neto, Aristóteles Góes Neto, Luciana V Barbosa, Mark J Guiltinan, Bryan A Bailey, Lyndel W Meinhardt, Julio CM Cascardo, Gonçalo AG Pereira

**Affiliations:** 1 Laboratório de Genômica e Expressão, Departamento de Genética e Evolução, Instituto de Biologia, Universidade Estadual de Campinas, CP 6109, 13083-970, Campinas – SP, Brazil; 2 Laboratório de Genômica e Biologia Molecular, Hospital A.C. Camargo, 01509-010, São Paulo – SP, Brazil; 3 HEMOCENTRO, Laboratório de Genoma e Hemoglobina, Universidade Estadual de Campinas, 13084-878, Campinas – SP, Brazil; 4 Departamento de Ciências Biológicas, ESALQ, USP, 13418-900, Piracicaba – SP, Brazil; 5 Laboratório de Genômica e Expressão Gênica, Departamento de Genética e Biologia Molecular, Universidade Estadual de Santa Cruz, 45650-000, Ilhéus – BA, Brazil; 6 Embrapa Recursos Genéticos e Biotecnologia, Parque Estação Biológica – PqEB – Av. W5 Norte, 70770-900, Brasília – DF, Brazil; 7 CEPLAC/CEPEC/SEFIT, 45600-970, Itabuna – BA, Brazil; 8 Laboratório de Biologia Molecular – Faculdade de Farmácia, Universidade Federal da Bahia, 40170-290, Salvador – BA, Brazil; 9 Laboratório de Pesquisa em Microbiologia (LAPEM), Departamento de Ciências Biológicas, Universidade Estadual de Feira de Santana (UEFS), 44031-460, Feira de Santana – BA, Brazil; 10 Laboratório de Biologia Molecular – Departamento de Biologia Geral, Instituto de Biologia, Universidade Federal da Bahia, 40170-290, Salvador – BA, Brazil; 11 Department of Horticulture, Pennsylvania State University, University Park, Chester, PA 16802, USA; 12 Sustainable Perennial Crops Laboratory, USDA-ARS, 10300 Baltimore Av, Bldg. 001, 18 Beltsville MD 20705-2350, USA

## Abstract

**Background:**

The basidiomycete fungus *Moniliophthora perniciosa *is the causal agent of Witches' Broom Disease (WBD) in cacao (*Theobroma cacao*). It is a hemibiotrophic pathogen that colonizes the apoplast of cacao's meristematic tissues as a biotrophic pathogen, switching to a saprotrophic lifestyle during later stages of infection. *M. perniciosa*, together with the related species *M. roreri*, are pathogens of aerial parts of the plant, an uncommon characteristic in the order Agaricales. A genome survey (1.9× coverage) of *M. perniciosa *was analyzed to evaluate the overall gene content of this phytopathogen.

**Results:**

Genes encoding proteins involved in retrotransposition, reactive oxygen species (ROS) resistance, drug efflux transport and cell wall degradation were identified. The great number of genes encoding cytochrome P450 monooxygenases (1.15% of gene models) indicates that *M. perniciosa *has a great potential for detoxification, production of toxins and hormones; which may confer a high adaptive ability to the fungus. We have also discovered new genes encoding putative secreted polypeptides rich in cysteine, as well as genes related to methylotrophy and plant hormone biosynthesis (gibberellin and auxin). Analysis of gene families indicated that *M. perniciosa *have similar amounts of carboxylesterases and repertoires of plant cell wall degrading enzymes as other hemibiotrophic fungi. In addition, an approach for normalization of gene family data using incomplete genome data was developed and applied in *M. perniciosa *genome survey.

**Conclusion:**

This genome survey gives an overview of the *M. perniciosa *genome, and reveals that a significant portion is involved in stress adaptation and plant necrosis, two necessary characteristics for a hemibiotrophic fungus to fulfill its infection cycle. Our analysis provides new evidence revealing potential adaptive traits that may play major roles in the mechanisms of pathogenicity in the *M. perniciosa*/cacao pathosystem.

## Background

*Moniliophthora perniciosa*, previously known as *Crinipellis perniciosa *(Singer) Stahel, is a hemibiotrophic basidiomycete (Tricholomataceae, Agaricales, Marasmiaceae) fungus that causes Witches' broom disease (WBD) in cacao (*Theobroma cacao *L.) [1-3]. WBD and frosty pod rot (FPR), caused by *Moniliophthora roreri*, are the most devastating diseases of cacao in the Americas [4]. Cacao production in southeastern Bahia, the main production area in Brazil, was severely affected by the introduction of WBD at the end of 1980's [5]. This disease damaged Bahian agribusiness, caused major social problems and has contributed to the degradation of the Atlantic Rainforest ("Mata Atlântica"). This is because cacao producing areas, typically, maintained old-growth native tree species as shade for the cacao plantations, which were converted to pasture [6].

The symptoms displayed by cacao plants during WBD parallel the hemibiotrophic development of *M. perniciosa *[7]. Briefly, the disease begins when fungal spores germinate and infect meristematic tissues, developing into monokaryotic biotrophic hyphae without clamp connections that slowly occupy the intercellular space. This stage of WBD is characterized by the emergence of hypertrophic and hyperplasic anomalous branches, and the formation of parthenocarpic fruits. Infected branches, known as "green brooms", grow without apical dominance, with a phototropic orientation and displaying epinastic leaves [7]. After two to three months the infected tissue turns necrotic (dry brooms) and the hyphae become saprotrophic with two nuclei per cell and clamp connections, invading the inter and intracellular spaces of the infected tissue [6,8]. This fungal species exhibits primary homothalism as its reproductive strategy [9]; thus, the change from the monokaryotic to the dikaryotic mycelium occurs without the prerequisite of mating between compatible individuals. After alternate wet and dry periods, basidiomes produced by the saprotrophic hyphae release basidiospores that are spread by wind or rain, thus completing the *M. perniciosa *life cycle [7].

The Witches' broom Genome Project  involving several Brazilian laboratories was initiated to increase the knowledge of this disease. The genome size, chromosomal polymorphism, genetic variability and the *M. perniciosa *mitochondrial genome have already been described [10-12]. Additionally, a biochemical study revealed the metabolic modifications that occur in cacao plantlets during WBD development [13]. Technical improvements have been achieved in the manipulation of cacao [14,15] and *M. perniciosa *[16,17]. One of the main bottlenecks in *M. perniciosa *research was solved with the development of the *in vitro *production of biotrophic-like cultures [18]. Necrotic inducing proteins expressed by *M. perniciosa *have been characterized [19], and the analysis of EST libraries and DNA microarrays have identified differentially expressed genes during its development [[20]; A.B.L. Pires *et al*., unpublished data] and for the interaction of the fungus with cacao [21,22]. Despite the substantial progression in understanding WBD, many questions remain unsolved, mainly those concerning the mechanisms controlling processes such as: (i) the fungal switch from biotrophism to saprotrophism; (ii) the drastic phenotypic alteration of cacao during disease development and (iii) the death of infected tissues. *In vitro *cultures in our laboratory demonstrate that this fungus has a great capacity to adapt to media containing different sources of carbon and nitrogen and it is able to grow in extremely nutrient-poor media. These results together with the fact that hemibiotrophic fungi, such as *M. perniciosa*, display complex lifestyles [23] suggest that this fungus has a significant genomic and transcriptomic plasticity that contributes to the successful pathogenic mechanisms expressed during its life cycle. In a recent review, Meinhardt *et al*., give a historical account and summarize the current state of knowledge about WBD [24].

Around two thirds of the known Basidiomycota species are included in the order Agaricales [25]. In addition to being an interesting group for carrying out developmental genetic studies concerning fungal development and reproduction, the Agaricales contain many important industrial species (i.e., edible mushrooms, fiber bleaching fungus), and species with unique lifestyles: saprophytes (i.e, wood-decaying fungus *Phanerochaete chrysosporium*), symbionts (i.e., ectomycorrhizal fungus *Laccaria bicolor*), leaf-litters decomposers (i.e., *Agaricus bisporus*) and root pathogens (i.e., *Armilaria mellea*). Interestingly, *M. perniciosa *and *M. roreri *are members of a group of Agaricales species that are able to infect aerial parts of plants, an uncommon characteristic among this Basidiomycota order.

Genome sequencing and analysis is an important strategy to obtain comprehensive information concerning the metabolism and development of organisms. The initial objective of the *M. perniciosa *Genome Project was to obtain a genome survey sequence using a whole shotgun strategy to provide genomic information for the WBD research community. Recently, the bioinformatics and genomic communities have been debating about the benefits and costs of finishing a complete genome as compared to applying a genome survey strategy [26-32]. It has been proposed that a two-fold genome sequence coverage is sufficient to support a high percentage of EST alignments and exon similarity matches [26,31]. Additionally, gene models resulting from a genome survey can be predicted accurately by the comparison with complete genomes of phylogenetically related organisms [26,31]. This strategy has been used for genomic surveys of dog (1.5× coverage) [31] and of the wine spoilage yeast *Dekkera bruxellensis *(0.4× coverage) [33]. Recently, the genomes of the basidiomycete species *Laccaria bicolor*, *Cryptococcus neoformans*, *Ustilago maydis, Coprinopsis cinerea *(*Coprinus cinereus*) and *Phanerochaete chrysosporium *have been determined and many additional Basidiomycota genome projects are ongoing or about to be released ; , which could provide a rich database for additional Basidiomycota genomic survey evaluations.

With the existence of several complete Basidiomycota genomes, and both *M. perniciosa *ESTs libraries, and a 1.9 × genome coverage, we decided to conduct a genome survey of *M. perniciosa *to obtain further information about this important phytopathogen. This report describes a survey of the genome sequences of *M. perniciosa*, with specific emphasis on the genes potentially involved in disease development such as a cytochrome P450 monooxygenases, transposable elements, putative plant defense elicitors, pathogenicity effectors, cell wall degrading enzymes, proteins related to methylotrophy and the biosynthesis of plant hormones by the fungus. The identification of such genes in the *M. perniciosa *genomic data lead us to hypothesize a connection between molecular processes involved in the growth phases of the fungus and the progression of WBD.

## Results and discussion

### Genome assembly and estimation of genome size

**Figure 1:**
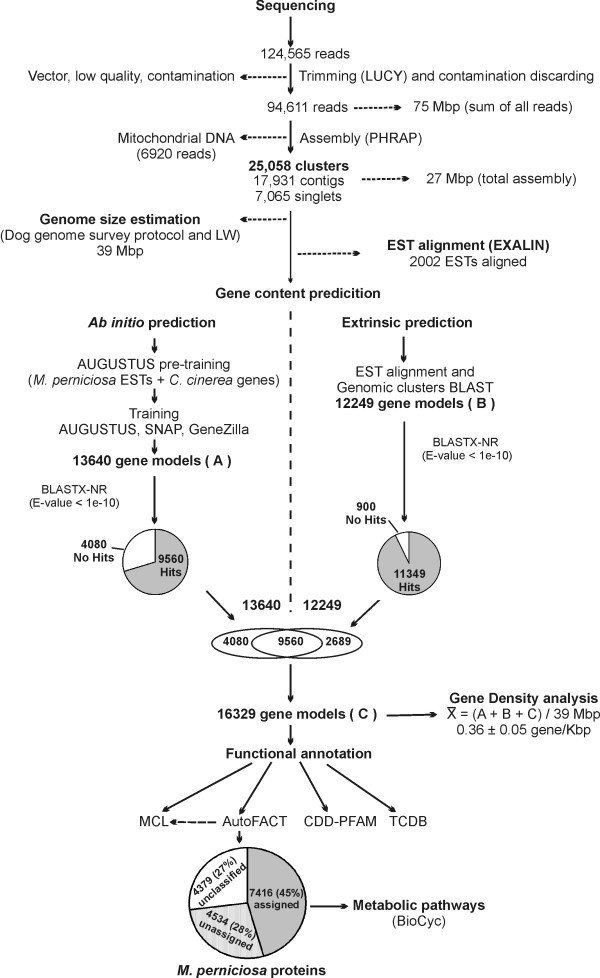
Flow diagram of bioinformatics procedures applied in *M. perniciosa *genome survey.

A diagram flow describing the bioinformatic procedures applied in the *M. perniciosa *genome survey are depicted in Figure 1. After sequencing, a total of 124,565 reads were obtained and assembled using the whole genome shotgun strategy. During the initial assembly process, a large contig including 6,920 reads was found. This contig was identified as the mitochondrial genome of the fungus (RefSeq NC_005927), which comprised approximately 6% of *M. perniciosa *sequences [12]. The remaining genome sequences were assembled resulting in 17,991 contigs and 7,065 singlets with average lengths of 1,300 bp and 455 bp, respectively. The largest contig consisted of 25,364 bp, and was formed by 513 reads. The sum of all reads was close to 75 Mbp and the total assembly consisted of 26.7 Mbp (Fig. 1).

Previous Feulgen-image analysis experiments estimated *M. perniciosa *genome size to be 32.98 ± 7.95 Mbp [10]. Due to the large error in this estimation we decided to assess the genome size using the genome estimation protocol established in the dog genome survey [31] (more details in Additional File 1). This analysis resulted in a genome size ranging from 38.7 to 39.0 Mbp, a value similar to the genome length of another fungus belonging the order Agaricales, *C. cinerea *(37.5 Mbp), and that agrees with the previous size estimated by Feulgen-image analysis [10].

The Lander Waterman theory (LW) [34] and its applications [35,36] were used to confirm the estimate of the *M. perniciosa *genome size. The theoretical values for the expected number of clusters (contigs + singlets), contigs, gaps, average cluster length and average gap length can be calculated using the LW theory from the effective average read length, the number of reads and the genome size as parameters. If the calculated values derived from the genome assembly were close to the theoretical values derived from LW calculations, then it is possible to confirm the estimated genome size. A summary of the results obtained from the assembly data and from the estimation using the LW theory is shown in Table 1. The experimental values agreed with the theoretical calculations, thus supporting our genome size estimation. According to LW analysis the average gap size was 413 pb. To have more information about the distribution of the gap size, we performed a comparison between a set of eukaryotic core proteins (generated by CEGMA pipeline [37]) and *M. perniciosa *contigs (See Additional file 1). Using this methodology, we detected that the average gap size was around 500 ± 300 bp, corroborating with LW average gap size estimative (413 bp). Considering that most of the gaps are around 500 bp and that *C. cinerea *average gene size is 1,678 bp, the majority of *M. perniciosa *genes or partial gene regions are likely to be included in the contigs. Corroborating this notion, we have found all genes encoding proteins of essential metabolic pathways such as Glycolysis, Gluconeogenesis, Pentose Phosphate pathway, and several others, which are available in the website  (see below).

**Table 1:** Comparison between assembly values and values calculated using Lander Waterman theory

	**Calculated from assembling**	**Calculated from LW theory**
# Clusters (contigs+singlets)	25,056	24,950
# Contigs	17,991	18,370
# Gaps	-	24,951
Average cluster size (bp)	1,065	1,152
Average gap size (bp)	-	413

In order to estimate the number of sequences misassembled due to repeat regions, we applied the integrated pipeline for assembly validation, called amosvalidate [38] (Further information in Additional file 1). This analysis resulted in 664 contigs with overrepresented regions totaling 1.1 Mbp. Multiplying the number of bases in overrepresented regions by over-coverage estimate resulted in 7.4 Mbp of repeat regions in the genome. Since reads from repetitive regions were eliminated from the estimation of the genome length using dog genome survey protocol (Additional file 1), this misassembling does not invalidates the genome size estimate.

### Gene content

The initial step to uncover the gene content of *M. perniciosa *was performed using the genomic sequences together with a library of 3,145 ESTs, previously annotated and partially published [20]. Genes were identified by comparing these two libraries using the program Exalin [39]. This analysis allowed us to identify expressed genes and intron structure. The result was 2,002 ESTs aligned to the genome contigs. Based on this EST-genome sequence alignment, the average intron length was estimated as 52 bp. This information was then used in the next steps of the process.

**Figure 2:**
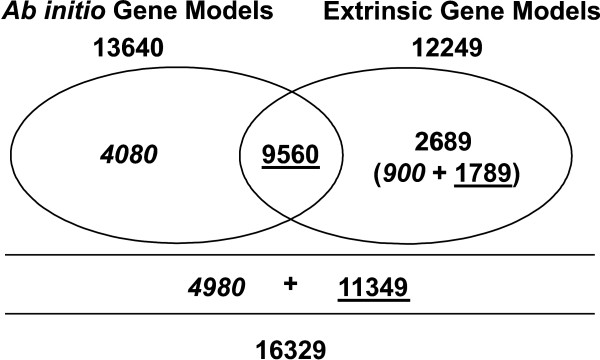
**Number of *M. perniciosa *gene models predicted by *ab initio *and/or extrinsic prediction methods.** Left ellipse: gene models predicted by *ab initio *methods. Right ellipse: gene models predicted by extrinsic methods. The intersection contains gene models detected by both methods. Underlined: number of gene models with BLASTX-NR E-value similarity ≤ 1e-10. In italics: number of gene models with BLASTX-NR E-value similarity > 1e-10.

A more detailed investigation of the gene content was carried out using a combination of *ab initio *(gene predictor programs) and comparative gene prediction (BLAST-EST sequence alignment). For *ab initio *gene prediction, we first applied the methodology described for the AUGUSTUS gene predictor [40]. This methodology essentially trains the AUGUSTUS program using a combination of sequences from the species of interest together with sequences of a phylogenetically related species, specifically with similarities in intron and exon length distributions. Using this approach, the coding content sensors (codon usage, GC content) are trained with sequences of the species of interest, and signal sensors (splice sites, TATA-box, polyadenilation sites, etc) are trained with the sequences of the related species. Sequences from *C. cinerea *were used in the training process; together with selected *M. perniciosa *ESTs with E-value in BLASTX-NR ≤ 1E-10. The sequences of the ESTs that aligned with proteins in the databank were concatenated, giving rise to a 240 Kbp sequence. Ten copies of this *M. perniciosa *EST concatamers (total of 2.4 Mbp) together with a dataset of genes from *C. cinerea *, comprised of 1.2 Mbp, were used to "pre-train" AUGUSTUS. The *M. perniciosa *predictions that came out of this pre-training were compared with the protein databank NR using BLASTp. The resulting predictions with similarities in the NR databank, and with a coverage ≥ 90%, were selected. After eliminating redundancies, 134 complete (containing the start codon and stop codon) and 1,136 partial (without the start codon and/or stop codon) *M. perniciosa *gene models were used to train AUGUSTUS [40] and two other gene predictors (SNAP [41] and Genezilla [42]). Predictions with less than 30 amino acids were eliminated. The remaining predictions were grouped into 19,932 overlapping clusters; that is, genomic regions covered by at least one prediction. The predictions in each overlapping cluster were ranked according to the criteria used by the Fungal Genome Initiative at the Broad Institute , with some adaptations. The *ab initio *gene finding pipeline generated 13,640 gene models, 9,560 of which contained significant similarity to GenBank sequences (Fig. 1 and Fig. 2).

The extrinsic prediction methodology consisted of a combination of genomic similarity searches (BLASTX) using contigs and singlets, with the alignment of *M. perniciosa *ESTs in the genomic clusters. 17,991 contigs and 7,065 singlets were submitted to similarity analysis in a databank containing BLASTX-NR plus *P. chrysosporium *proteins. The genomic regions containing homologues in this databank were selected and exon-intron boundaries were determined. We also selected the alignments between ESTs and genomic clusters. Then, we evaluated if there was a superposition of BLAST alignments and EST alignments. After this analysis, the extrinsic prediction methodology revealed 12,249 gene models. Most of these gene models (9,560) were also predicted by the *ab initio *gene predictor programs. Of the 2,689 remaining gene models not predicted by the gene predictor programs, 1,789 presented significant similarity to sequences deposited in the GenBank (E-value ≤ 1E-10) and 900 did not have any significant similarity in the GenBank (E-value > 1 E-10) (Fig. 1 and Fig. 2). One of the reasons why these genes were not detected by the gene prediction programs could be the presence of low quality sequences that may have lead to frameshifts, thus making them impossible to detect by these programs. The total number of gene models obtained using both approaches, *ab initio *and extrinsic predictions, is 16,329. Assuming that the average contig length is 1.3 Kbp and the average gap length is 413 bp, it is possible that this total number of gene models (16,329) represents an overestimation. For instance, in our assembly a gene with > 2 Kbp (protein ~700 aa) could be represented by two gene models, with each one in different contigs, which suggests a possible redundancy in this gene model prediction. Thus, it is very likely that the real number of gene models will be less and closer to the *ab initio *or extrinsic predictions separately. However, to obtain the maximum amount of information, we decided to evaluate the results of similarity searches in GenBank (BLASTX-NR) for all 16,329 gene models. As a result, 69.5% (11,349) of the gene models showed significant sequence similarity (E-value ≤ 1e-10) to GenBank proteins. The remaining 30.5% (4,980), designated as "no-hits", were sequences with lower E-value scores (E-value > 1e-10). Of these gene models 4,080 were predicted with the *ab initio *gene predictors and 900 with the extrinsic predictions (Fig. 1 and Fig. 2).

As expected, BLASTX analysis against available complete fungal genomes showed that *M. perniciosa *gene models present higher similarity to genes from Basidiomycota (*L. bicolor*, *C. cinerea *and *P. chrysosporium*) than those from Ascomycota (Table 2).

**Table 2:** *Moniliophthora perniciosa *predicted gene comparisons

**Organism Comparison**	Genes with* BLASTX hits	**Genes with Top BLASTX Hits**
*Laccaria bicolor*	9910 (60.6%)	4811 (29.27%)
*Coprinopsis cinerea*	10649 (65.1%)	3281 (19.97%)
*Phanerochaete crhysosporium*	9311 (57.0%)	2021 (12.30%)
*Fusarium graminearum*	5904 (36.1%)	57 (0.35%)
*Magnaporthe grisea*	5645 (34.5%)	39 (0.2%)
*Ustilago maydis*	5488 (33.6%)	42 (0.26%)
*Cryptococcus neoformans*	5454 (33.4%)	78 (0.47%)
*Neurospora crassa*	5127 (31.4%)	16 (0.10%)
*Saccharomyces cerevisiae*	3296 (20.2%)	4 (0.02%)
All NR database	11349 (69.5%)	_

* – Each *M. perniciosa *gene model was compared to all the proteins from the organisms listed in the table. BLASTX score was defined as 1e-10

In order to find groups of similar proteins in our dataset, we applied a Markov Clustering (MCL) algorithm [43] to the *M. perniciosa *gene models. Although the output by this method is not highly reliable, they correlate well with "real" gene families and can be applied efficiently to cluster large quantities of genes in a high throughput fashion [44,45]. MCL also helped to the assemble genes without similarity in the GenBank into gene families, which are described in the following sections.

**Figure 3:**
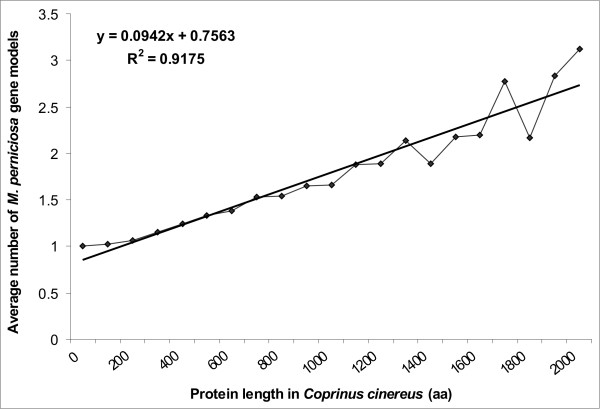
Correlation between the average number of *M perniciosa *gene models and the length of *C. cinereus *proteins.

Those gene models that were not grouped into gene families by the MCL algorithm have been compared with the genome of *C. cinerea *in order to discard those genes with parts of their sequences present in more than one contig. In order to perform this comparison (TBLASTN) we assume that the length distribution of *M. perniciosa *proteins is similar to those from *C. cinerea*. In this comparison we evaluated similar genes according to the length of the protein (Fig. 3). This comparison showed that there is a clear correlation between complete genes in *C. cinerea *and *M. perniciosa *gene models coding for proteins smaller than 300 aa. However, for larger proteins more than one *M. perniciosa *gene model showed similarity to a single *C. cinerea *protein, thus confirming the overestimation of the number of gene models. Given the fact that the genome sequence is incomplete, there is high probability that we are predicting more than one gene model per gene.

In order to have a measurement of the overestimation and use it to correct our database, the corresponding relationship between the protein size and the number of gene models was calibrated with the genome of *C. cinerea *using the gene models unlinked to gene families identified by the MCL algorithm (Fig. 3). A linear regression formula was used to correct for the overestimation according to the protein size. For example, proteins smaller than 300 amino acids will have only one model representing the gene, while larger proteins will have one model and a fraction of a second model representing its gene. This fraction represents the overestimation of the number of gene models.

**Figure 4:**
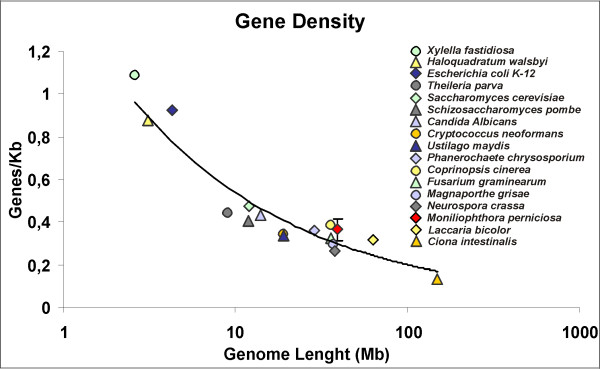
**Correlation of various organisms' genome size and number of genes (Gene Density).** Error bar in *M. perniciosa *data point depicts the Standard Deviation (SD ± 0.05) of Gene/Kbp ratio using *ab initio *predicitions, extrinsic predictions and the sum of both predictions (see text).

In order to estimate the gene density in *M. perniciosa*, and deal with the overestimation evident by the previous analysis, we averaged the total number of gene models obtained by the different predictions: *ab initio *predictions (13,640), extrinsic predictions (12,249) and total number of gene predictions (16,329). This resulted in 14,072 gene predictions that were divided by the 39 Mbp estimated genome size, which gave a gene density of approximately 2.77 ± 0.37 Kbp/gene or 0.36 ± 0.05 gene/Kbp for the genome of *M. perniciosa*. Figure 4 depicts the gene density comparison between *M. perniciosa*, two eubacteria (*Xyllela fastidiosa *and *Escherichia coli*), one archea (*Haloquadratum walsbyi*), an apicomplexan (*Theileria parva*), a primitive chordate (*Ciona intestinalis*) and a series of fungal genomes, and this showed that our gene density evaluation is in agreement with gene density data of other filamentous fungi [46].

The overall *M. perniciosa *genomic features are summarized in Table 3. The genomic clusters of *M. perniciosa *were submitted to NCBI (GenomeProject ID 28951, Locus-tag prefix MPER) and the Whole Genome Shotgun project has been deposited at DDBJ/EMBL/GenBank under the project accession ABRE00000000. The version described in this paper is the first version, ABRE01000000.

**Table 3:** *Moniliophthora perniciosa *genome survey features

**Genome survey features**	**Values**
Contigs	17,991
Contigs size (average)	1.3 Kbp
Singlets	7,065
Singlets size (average)	455 bp*
Sum of all reads	75 Mbp
Assembly size	26.7 Mbp
Estimated genome size	39 Mbp
Coverage	1.92×
Gene models	
*Ab initio *gene models	13,640
Extrinsic gene models	12,249
Total gene models	16,329
Gene density	2.77 Kbp/gene (SD ± 0.39)
	0.36 gene/Kbp (SD ± 0.05)
Average gene size	651.4 bp
GC content overall	47.7
GC content coding	49.7
GC content non coding	46.8
Intron size (mode)	52 bp
Intron size (average)	63 bp
Exon size (mode)	60 bp
Exon size (average)	166 bp
AutoFACT annotation	
Classified (Hits)	11,950 (73%)
Assigned (Conserved expressed proteins)	7,416 (45%)
Unassigned (Conserved hypothetical proteins)	4,534 (28%)
Unclassified (No Hits)	4,379 (27%)

* – Singlet size after LUCY quality trimming (phred ≥ 16).

### Overall Functional Annotation and Metabolic maps

The number of gene models used for the functional annotation and building of the metabolic maps was 16,329, which represents the total number of gene models obtained using *ab initio *and extrinsic predictions. Although this number represents an overestimation of the real number of genes (see above), it was used to maximize the information acquired from these sequences. All data obtained in the gene families and protein domain analyses were corrected according to the normalization procedure developed from the gene models not included in any of the MCL families based on comparisons with the *C. cinerea *genome (see above).

The program AutoFACT [47], an automated annotation tool, was used to evaluate the putative functions of *M. perniciosa *gene models. This program determines the most informative functional description by combining multiple BLAST reports from a number of user selected databases, and provides a consensus result [47]. AutoFACT classifies proteins as "classified proteins", which can be "assigned" (containing similarity to proteins with annotated function) or "unassigned" (containing similarity to proteins with unknown function), and "unclassified proteins" (without similarity to any other protein in databanks). Using this software, 73% of gene models (11,950) were annotated as previously classified proteins. From these 7,416 (45%) were assigned proteins, and 4,534 (28%) were classified as unassigned proteins, which can be interpreted as a class of conserved hypothetical proteins (Table 3). The remaining 4,379 gene models (27%) were considered as unclassified proteins. The number of gene models with E-value scores > 1e-10 obtained by BLASTX-NR (no-hits, see previous section) was 4,980 and represented 30.5% of the total number of models. The result obtained with AutoFACT increased the number of classified proteins by 3.5% (Fig. 1; Table 3). This indicates that the use of domain classification databases is helpful in the identification and annotation of gene models.

Additionally, we correlated the AutoFACT annotation with the gene families assembled by the MCL algorithm. As depicted in Table 4 and additional file 2, MCL data indicated that the cytochrome P450 monooxygenase family had the largest number of *M. perniciosa *members, followed by a gypsy-like retrotransposon gene family, and a gene family that has similarity to a *C. cinerea *hypothetical protein (EAU86912.1) and to a shitake mushroom *Lentinula edodes *EST (EB016963). This new gene family appears to be related to specific developmental traits of the Agaricales. Also found were new gene families with unknown functions based on their lack of homology with GenBank sequences (Additional file 2; Additional File 3 – Worksheet Unknown Gene Families). These families could possibly be linked to *M. perniciosa *physiological characteristics.

**Table 4:** MCL analysis of *M. perniciosa *gene models

**MCL family ID**	**#members^a^**	**Norm Factor^b^**	**Norm #members^c^**	**Manual annotation^d^**
1	175	1.24987	140	Cytochrome P450 monooxygenase
2	143	1.82632	78	Gypsy like-retrotranspon
4	84	1.35205	62	Conserved hypothetical protein
3	84	1.46889	57	Alcohol oxidases and GMC oxidoreductase
5	82	1.26479	65	Gypsy like-retrotranspon
6	76	1.57535	48	Serine threonine kinase
7	64	1.54312	41	Conserved hypothetical protein
8	59	1.54180	38	Conserved hypothetical protein
9	57	1.46370	39	Proteins containing WD-40 and NACHT domain
10	55	-	-	*No hits *protein family
11	52	1.34190	39	Cytochrome P450
12	50	1.52169	33	Serine threonine kinase
13	46	1.18098	39	Conserved hypothetical protein
14	43	1.21555	35	Cytochrome P450
15	41	1.45275	28	Carboxylesterase/lipase
16	39	1.67524	23	Gypsy like-retrotranspon
17	38	1.26598	30	Hexose transporter
19	35	1.11159	31	MFS transporter
18	35	1.43881	24	Splicing factor
20	32	1.17902	27	Laccase – Mulitcopper oxidase

a – Number of gene models present in each family

b – Normalization Factor used to normalize the number of gene models present in each family (see methods)

c – Normalization number of gene models present in each family

d – Manual annotation of MCL families according to BLAST similarity analysis

A comparison of the gene models with the CDD-PFAM databank [48] was performed to obtain information about protein domains present in *M. perniciosa *proteins. The data obtained were normalized using the procedure described above. Cytochrome P450 monooxygenase was the most prevalent protein domain assigned in *M. perniciosa *(gnl|CDD|40168), followed by protein kinases (gnl|CDD|40170), sugar transporters (gnl|CDD|40184), short chain dehydrogenases (gnl|CDD|40206) and carboxylesterases/lipases (gnl|CDD|40235) (Table 5; Additional File 4). The prevalence of Cytochrome P450 monooxygenase domains in the genome agrees with the results of MCL analysis. CDD-PFAM analysis was also used to evaluate the protein domains in other genome fungi (Additional File 4). The comparisons between *M. perniciosa *and the other fungi analyzed are described below.

**Table 5:** Top 20 CDD-PFAM domains in *M. perniciosa *proteins

**CDD – ID^a^**	**Domain PFAM^b^**	**#Hits^c ^Domains**	**%Hits^d ^PTN**	**Rank^e^**	**Norm Factor**	**# Hits^g ^Domains Norm**	**%Hits^h ^PTN Norm**	**Rank^i ^Norm**
gnl|CDD|40168	pfam00067, Cytochrome P450	256	1.57%	1	1.3565	188.71427	1.15%	1
gnl|CDD|40170	pfam00069, Protein kinase	141	0.86%	2	1.6059	87.80184	0.54%	2
gnl|CDD|40184	pfam00083, Sugar (and other) transporters	89	0.54%	3	1.6902	52.65651	0.32%	3
gnl|CDD|40206	pfam00106, adh_short, short chain dehydrogenase	84	0.51%	4	1.7141	49.00442	0.30%	4
gnl|CDD|40235	pfam00135, COesterase, Carboxylesterase	57	0.35%	5	1.4738	38.67603	0.24%	5
gnl|CDD|40207	pfam00107, ADH_zinc_N, Zinc-binding dehydrogenase	43	0.26%	8	1.2398	34.68221	0.21%	6
gnl|CDD|40813	pfam00732, GMC_oxred_N, GMC oxidoreductase	52	0.32%	6	1.5402	33.76192	0.21%	7
gnl|CDD|40345	pfam00248, Aldo_ket_red, Aldo/keto reductase family	41	0.25%	9	1.2881	31.83088	0.19%	8
gnl|CDD|41245	pfam01185, Hydrophobin, Fungal hydrophobin	26	0.16%	21	0.8460	30.73119	0.19%	9
gnl|CDD|40253	pfam00153, Mito_carr, Mitochondrial carrier protein	38	0.23%	12	1.3890	27.35860	0.17%	10
gnl|CDD|40748	pfam00665, rve, Integrase core domain	50	0.31%	7	1.8943	26.39449	0.16%	11
gnl|CDD|40493	pfam00400, WD40, WD domain, G-beta repeat	35	0.21%	13	1.5151	23.10130	0.14%	12
gnl|CDD|40179	pfam00078, RVT, Reverse transcriptase	39	0.24%	11	1.7620	22.13448	0.14%	13
gnl|CDD|45101	pfam05199, GMC_oxred_C, GMC oxidoreductase	28	0.17%	16	1.2741	21.97654	0.13%	14
gnl|CDD|40588	pfam00501, AMP-binding, AMP-binding enzyme	40	0.24%	10	1.8537	21.57819	0.13%	15
gnl|CDD|41412	pfam01360, Monooxygenase, Monooxygenase	25	0.15%	22	1.2253	20.40382	0.12%	16
gnl|CDD|40107	pfam00005, ABC_tran, ABC transporter	33	0.20%	14	1.7198	19.18851	0.12%	17
gnl|CDD|40367	pfam00270, DEAD, DEAD/DEAH box helicase	30	0.18%	15	1.5947	18.81230	0.12%	18
gnl|CDD|40419	pfam00324, AA_permease, Amino acid permease	26	0.16%	20	1.4641	17.75798	0.11%	19
gnl|CDD|41783	pfam01753, zf-MYND, MYND finger	25	0.15%	23	1.4288	17.49737	0.11%	20

a – Number of CDD entry

b – Number and description of

PFAM domain

c – Number of gene models containing each CDD-PFAM domain

d – Percentage of gene models containing each CDD-PFAM domain in relation to total number of *M. perniciosa *gene models (proteins).

e – Non-normalized CDD-PFAM ranking

f – Factor used to normalize the number of gene models containing each CDD-PFAM domain (see methods)

g – Normalized number of gene models containing a CDD-PFAM domain

h – Percentage of normalized number of gene models containing each CDD-PFAM domain in relation to total number of *M. perniciosa *gene models (proteins)

i – Normalized *M. perniciosa *CDD-PFAM ranking

A hypothetical metabolic map of *M. perniciosa *was built using BioCyc [49]. This analysis allowed us to annotate 235 metabolic pathways. These include 1358 enzymatic reactions incorporating a total of 2139 enzymes . A comparison of this metabolic map with the fungal model *Saccharomyces cerevisiae *S288C (documented with 132 pathways, 925 enzymatic reactions, and 675 enzymes – ) showed that *M. perniciosa *has more metabolic pathways than *S. cerevisiae*; a result that corresponds to the smaller genome size of *S. cerevisiae *and possibly the more complex lifestyle of *M. perniciosa*. Interestingly, *M. perniciosa *has a higher number of reactions involving O_2_, CO_2_, H_2_O_2_, and NAD(P)+/NAD(P)H than *S. cerevisiae*, suggesting a greater capacity to use and deal with oxidation-reduction reactions (Additional File 5 – worksheet compounds). We also detected reactions with farnesyl pyrophosphate and dimethylallyl-diphosphate, which are involved in the biosynthesis of the secondary metabolites such as isoprenoids and indoles. Moreover, according to BioCyc analysis, *M. perniciosa *has more amino acid catabolic pathways, alternative carbon sources degradation and biosynthesis routes and C1 compounds (i.e., methanol) utilization and assimilation reactions than *S. cerevisiae *(Additional File 5 – worksheet pathways). These pathways are under manual annotation and will be published on BioCyc web page. The existence of these pathways in *M. perniciosa *suggest ecological and physiological adaptations to environmental stresses; to competition present in its native habitat in the Amazon Basin, and to traits that enable it to colonize cacao and trigger WBD.

### Detoxification and general resistance mechanisms: cytochrome P450 monooxygenases, efflux transporters and anti-oxidative apparatus

**Figure 5:**
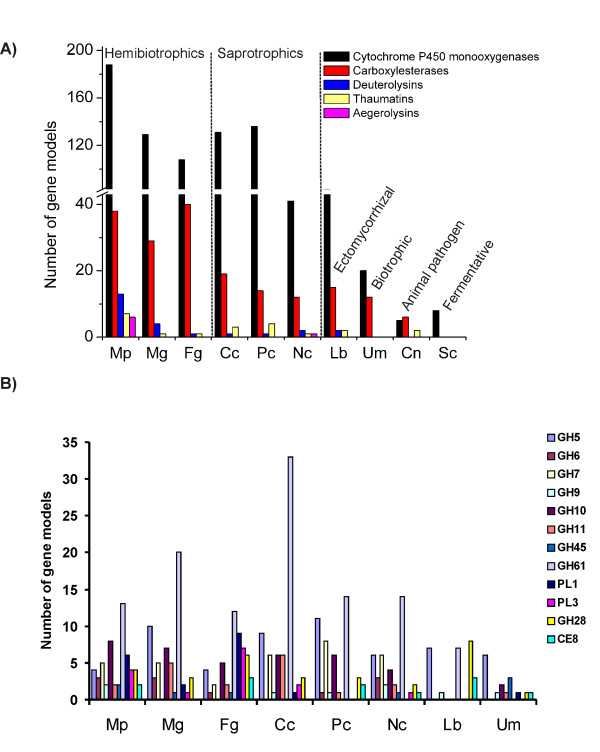
**Comparison of *M. perniciosa *protein families with other fungi**. (A) comparison between cytochrome P450 monooxygenases, carboxylesterases, deuterolysins, thaumatins and aegerolysins; (B) comparison of plant cell wall degrading enzymes from fungi that interact with plants. Mp = *Moniliophthora perniciosa*, Lb = *Laccaria bicolor*, Cc = *Coprinopsis cinerea*, Pc = *Phanerochaete chrysosporium*, Um = *Ustilago maydis*, Mg = *Magnaporthe grisea*, Cn = *Cryptococcus neoformans*, Fg = *Fusarium graminearum*, Nc = *Neurospora crassa*, Sc = *Saccharomyces cerevisiae*. The legend at the right refers to the nomenclature of plant cell wall degrading enzymes according to CAZy .

Based on CDD-PFAM and MCL analyses gene members of the cytochrome P450 monooxygenase superfamily are prevalent in the genome of *M. perniciosa *(Tables 4 and 5; Additional File 3 – Worksheet P450; Additional File 4). Cytochrome P450 monooxygenases play a role in hydroxylation and oxidation processes involved in biosynthesis and degradation of different compounds [50]. Therefore, a large number of gene models similar to cytochrome P450 monooxygenases suggest a significant capacity for synthesis of secondary metabolites, such as hormones or toxins, and for detoxification. Among the fungi analyzed, *M. perniciosa *has the highest number of cytochrome P450 monooxygenase genes (188 gene models) representing 1.15% of the gene models (Fig. 5A). Saproprotrophic basidiomycetes *P. chrysosporium *and *C. cinerea *and the hemibiotrophic ascomycetes *Magnaporthe grisea *and *Fusarium graminearum *also have more than one hundred cytochrome P450 monooxygenase genes representing between 1.25% and 0.9% of their gene models (Figure 5A; Additional File 4). The basidiomycete *L. bicolor *(an ectomycorrhizal fungus) and the ascomycete *Neurospora crassa *(a fire-scoured landscape colonizer) follow with fewer genes. *U. maydis *(a biotrophic pathogen), *C. neoformans *(an animal pathogen) and *S. cerevisiae *(a fermentative fungus) have the fewest cytochrome P450 monoooxygenase genes of the fungi compared in this study (Fig. 5A; Additional File 4). This analysis clearly demonstrates the prevalence of cytochrome P450 monooxygenases in saprotrophic and hemibiotrophic fungi, which have to hydrolize complex wood polymers and deal with a highly oxidative environment. As discussed by Gonzalez and Nebert [50], cytochrome P450 monooxygenase polymorphisms may be the product of the "molecular warfare" that occurs during the co-evolution of preys and predators, which produce toxins and detoxifying genes, respectively. This logic can be extrapolated to the plant-fungus interaction, and in that sense, we believe that the plethora of cytochrome P450 monooxygenases in *M. perniciosa *may be critical to detoxification and environmental adaptation as well as for disease development.

Another set of proteins related to detoxification processes are the efflux transporters. Similarity searches in the Transport Classification Database (TCDB – ) [51] which compare the genome of *M. perniciosa *with other fungal genomes (Additional File 3 – Worksheet Functional annotation and Worksheet Transporters) verified an extended set of efflux transporters from classes 3.A.1 (ABC superfamily) and 3.A.3 (P-type ATPase Superfamily). The majority of ABC transporters identified in *M. perniciosa *belong to the Pleiotropic Drug Resistance (PDR) family and the ABC Conjugate (ABCC) Transporter family. These proteins have been associated with fungal pathogenesis [52] and heavy metal resistance [53]. The members of P-type ATPase superfamily in *M. perniciosa *include phospholipid translocating ATPases family and fungal ENA-ATPases transporters, which are involved with the efflux of excessive Na^+^, and especially K^+^, encountered by fungi during colonization of plants [54].

During the plant defense, reactive oxygen species (ROS) are produced to limit the pathogen invasion [55]. However, pathogenic fungi produce antioxidant enzymes that enable them to neutralize host ROS. *M. perniciosa *contains a myriad of O_2_^- ^and H_2_O_2 _decomposing enzymes such as superoxide dismutases, catalases, peroxiredoxins, glutathione-system, thioredoxin-system enzymes and manganese dependent peroxidases (Additional File 3 – Worksheet Functional Annotation). Therefore, this fungal genome harbors a complete ROS detoxification system. Reports indicate that H_2_O_2 _favors necrotrophic pathogens infection [56,57]. Furthermore, *M. perniciosa *produces calcium oxalate crystals (COC) [58], and a cacao susceptible genotype accumulates COC during *M. perniciosa *infection, followed by a programmed cell death (PCD) [59]. The degradation of COC produces carbon dioxide and H_2_O_2_, suggesting that COCs can be important to necrotrophic mycelia development. In addition, oxalate chelates Ca^2+^, an important secondary plant defense messenger and a key cross-linker of pectin in the middle lamella pectin [60], and was found to be a trigger of PCD in plants [61]. These findings suggest that oxalate favors *M. perniciosa *infection by disorganizing plant defense and plant cell wall structure, by facilitating the action of fungal pectinases and possibly triggering PCD in the later stages of WBD [59].

### Genome variability: Mating-type genes and transposable elements

Seven *M. perniciosa *gene models were found to be similar to pheromone receptors (Additional File 3 – Worksheet Functional Annotation). As a primary homothallic fungus, *M. perniciosa *does not use its mating type system to outcross, but probably to promote the formation of clamp connections, hyphae dikaryotization and for the expression of pathogenicity genes as in *U. maydis *[62]. Previous reports have indicated that *M. perniciosa *exhibits high genetic variability at the molecular level [11,63,64]. This level of variability may be the reason *M. perniciosa *overcomes resistant genotypes of cacao, such as Scavina 6 [65]. Furthermore, it has been postulated that the genome variability found in homothallic *M. perniciosa *may be due to transposable elements (TEs) and ectopic recombination guided by the numerous copies of these elements found in the genome [10]. The fact that retrotransposons were identified in EST libraries and differentially expressed during *M. perniciosa *development [20], indicates that they are active elements, which could contribute to genetic variability. Among the *M. perniciosa *TE families, Gypsy-like retrotransposons were the most abundant, followed by *Copia*-like retrotransposons (Tables 4 and 5; Additional File 2; Additional File 3 – Worksheet Transposons). TEs similar to *P. chrysosporium *Copia-like elements were found in *M. perniciosa *genome inserted within putative cytochrome P450 monooxygenase genes [66]. Curiously, they were also inserted in a *P. chrysosporium *cytochrome P450 monooxygenase subfamily (Additional file 2 – family 255; Additional File 3 – Worksheet P450), suggesting that these TEs may have a common ancestral origin in Basidiomycota. Retroelements of the tyrosine recombinase (YR) order [67] and DNA transposons (class II transposons) from CACTA [68], hATC [69,70] and Tc/Mariner [71] superfamilies were also found. Finally, a previously described *Boto *DNA transposon [M.V. Queiroz *et al*., unpublished data] from the PIF/IS5 superfamily [72] was also identified in a MCL family (Additional File 2 – family 251; Additional File 3 – Worksheet Transposons).

### Plant Hormonal Disarrangement: Fungal genes related to plant hormones biosynthesis

There is growing evidence of phytohormones being produced by pathogens during some infective processes [73]. For instance, the production of gibberellins (GA); hormones involved in the regulation of stem elongation, seed germination, flowering and fruit maturation; have been identified in phytopathogenic bacteria and fungi that cause overgrowth symptoms, such as *Giberella fujikuroi *and *Sphaceloma manhiticola *[74,75]. A search for homologues of the fungal specific bi-functional ent-kaurene synthase (CPS/KS) responsible for the two-step cyclation of GGDP in fungi [76] identified gene models similar to the N-terminal domain of *G. fujikuroi *CPS/KS but did not detect any sequence similar to the C-terminal domain of this protein in *M. perniciosa*. Another gene model similar to CPS/KS that lacks the C-terminal domain was found in the *Aspergillus niger *genome (AM270241.1). Genes similar to GA-4 desaturase and GA oxidases (cytochrome P450), part of a GA biosynthesis gene cluster present in *G. fujikuroi *and *Phaeosphaeria *sp. were detected (Additional File 3 – Worksheet Functional Annotation). Reinforcing our data mining discover, is the fact that a gibberellin-like compound was detected in basidiospores of *M. perniciosa *[77]. We can theorize that the production of GA by *M. perniciosa *may confer the hyperplasic phenotype of the green broom that resembles stem hyper-elongation caused by GA-producing phytopathogens.

**Figure 6:**
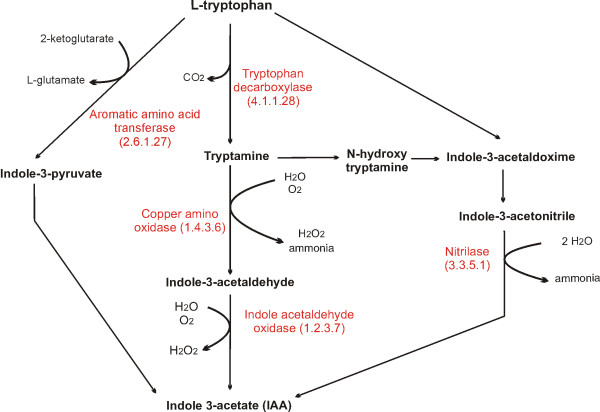
**Indole-3-acetate (IAA) biosynthesis pathways. ***M. perniciosa *gene models are annotated in red.

Another interesting discovery in the *M. perniciosa *genome is the presence of genes encoding enzymes of two biosynthetic pathways of indole-3-acetic acid (IAA), the most abundant natural plant auxin. We found a gene similar to plant nitrilases (E.C 3.5.5.1) which catalyzes the direct conversion of indole-3-acetonitrile into ammonia and IAA. Additionally, potential genes for the IAA-producing tryptamine pathway (one tryptophan decarboxylase, copper amine oxidases and a hypothetical indole-acetaldehyde oxidase) were found (Fig. 6; Additional File 3 – Worksheet Functional Annotation). Furthermore, a manual annotation of EST libraries reveal the presence of an aromatic amino acid aminotransferase, which could make part of Indole-3-pyruvate IAA biosynthetic pathway (Fig. 6). IAA regulates many plant biological processes including cell elongation and fruit ripening. Recently, the presence of IAA in *M. perniciosa *basidiocarps was reported [78]. IAA is produced by other fungal phytopathogens such as biotrophics *U. maydis *and *Taphrina deformans *and hemibiotrophic *Colletotrichum gloeosporioides *sp. [79-81], and induces filamentation and invasive growth in *S. cerevisiae *[82]. Curiously, both Gibberellin and IAA induce fruit parthenocarpy and act synergistically in plant organ expansion [83], both of which are traits of WBD.

Cacao plantlets inoculated with *M. perniciosa *spores emit higher levels of ethylene during the late stages of infection than non-inoculated plants [13]. The plant hormone ethylene stimulates elongation at low concentrations, and senescence, fruit ripening, and epinasty at high doses [84]. Auxins stimulate the synthesis of ethylene [85], which together with gibberellins have integrated actions in plant cell death [86] and stem elongation during phytochrome-mediated shade avoidance, a phenomenon that occurs in response to the low red to far-red light ratios (R:FR) under dense canopies [87]. We hypothesize that in a dense and shaded environment, such as a cacao plantation, the low R:FR ratio effects can be increased by the action of the aforementioned phytohormones, explaining the shade avoidance, hypertrophy, and elongation of green brooms. The presence of genes related to plant hormones production in *M. perniciosa *supports previous data [77,78] and suggests that this fungus can influence the plant metabolism and defense, by altering hormonal balance during infection [73,88].

### Pathogenicity: Fungal effectors and pathogenicity associated proteins

As a pathogen that colonizes the plant apoplast during its biotrophic stage, *M. perniciosa *may release elicitor or effector proteins into the extracellular medium, which in turn could evade or suppress the plant defense response. Throughout WBD, *M. perniciosa *produces proteins with the potential to kill plant cells, thereby releasing their contents, which are absorbed by the fungus during its saprotrophic stage. Thus, *M. perniciosa *uses a varied arsenal of effector proteins in order to complete its infection cycle. Although some phytopathogenic fungi deliver effector proteins into the cytoplasm by means of haustoria [89,90] this type of structure is absent in *M. perniciosa*. Therefore it is possible that this fungus secretes these proteins into the apoplast, as has been described for other fungi [91]. Effector proteins that are recognized by plant resistance (R) proteins are known as *avr *proteins. No orthologues to the known Ascomycota *avr *genes, not even the *U. maydis *genes contained in "biotrophic clusters" [92], were found in the genome of *M. perniciosa*. However, *ab initio *gene prediction with peptide signal analysis revealed 70 "no hits" small proteins containing secretion signals and at least two cysteines (see methods, Additional File 6), which is a common trait of many proteins that are delivered into the host apoplast by phytopathogens [93]. In order to validate 22 selected *ab initio *predictions, we conducted RT-PCR using RNA from *M. perniciosa *saprotrophic mycelia. We validated the expression of 13 gene models out of 22 tested, all of which contained secretion signals (Additional File 7 – Primers No Hits Cys protein). Possibly, the gene models that were not confirmed by RT-PCR are expressed in other developmental stages of the fungus (basidiome, spore, etc.). Additional File 8 depicts the amplification of three of these genes. Whether these proteins play a role in the pathogenicity of *M. perniciosa *or in elicitation of cacao defense remains to be elucidated.

*M. perniciosa *contains Necrosis and Ethylene inducing proteins (NEPs) and cerato platanins [19,94], which can act in conjunction with a series of proteinases, hemolysin-like proteins and carboxylesterases/lipases found in the genome (Additional File 3 – Worksheet Functional Annotation). These proteins appear to be part of the destructive arsenal of *M. perniciosa*. The most abundant proteinases in the genome of *M. perniciosa *are deuterolysins, a type of fungal metalloproteinases that are similar to bacterial thermolysin [95]. Compared to other fungi indicates that *M. perniciosa *has a deuterolysin expansion (13 gene models; Fig. 5A; Additional File 4), suggesting an important role for these proteinases during this fungus development.

We also identified a gene family similar to agaricales *Pleurotus ostreatus *and *Agrocybe aegerita *hemolysin-like aegerolysins. These proteins have cytolitic properties [96] and seems to play an important role at the initial phase of fungal fruiting by making the fungal membranes permeable during cell signaling [96]. *M. perniciosa *genome contain a family of aegerolysins (6 aegerolysins; Fig. 5A; Additional File 4). None of the other agaricales fungi analyzed (*L. bicolor*, *C. cinerea *and *P. chrysosporium*) contain these proteins. Even though we could not assess the genomic data of the basidiomycete containing aegerolysins, we suggest that the diversification of these proteins in WBD causative agent indicates their importance in *M. perniciosa *development or even in fungi defense and infective process.

Carboxyesterases and lipases are overrepresented in *M. perniciosa *(Tables 4 and 5; Additional Files 2 and 4). According to CDD-PFAM, *M. perniciosa *have 38 gene models annotated as carboxylesterases, approximately twice the number of such proteins in other basidiomycete (ectomycorrhizal *L. bicolor *(15); saprobes *C. cinerea *(19) and *P. crhysosporium *(14); and biotrophic *U. maydis *(12); Figure 5A; Additional File 3); while the hemibiotrophic ascomycete *F. graminearum *and *M. grisea *have 40 and 29 carboxylesterases, respectively. We postulate that the great number of carboxylesterases and lipases in *M. perniciosa*, *F. graminearum *and *M. grisea *is related to their hemibiotrophic lifestyle. In fact, these enzymes are induced during carbon and nitrogen starvation [97], and cell wall degradation [98], two events that occur during hemibiotrophism.

Other genes associated with the plant-pathogen interaction found in our analysis were similar to the SCP-like superfamily proteins, which comprise pathogenesis related (PR) proteins of family 1 (PR-1). Additionally, gene models similar to PR-5/thaumatin superfamily were also detected in the *M. perniciosa *genome (Additional File 3 – Worksheet Functional Annotation). PR proteins are well described as associated with defense reactions in plants against various pathogens [99]. For instance, transgenic plants overexpressing PR-1 proteins were more resistant to oomycete infection [100] and some Thaumatin-like proteins (TLPs) have β-glucanase activity, inhibit xylanase and have antifungal properties [101-103]. Recently, proteins similar to PR-1 and thaumatin have been characterized in animals and fungi [104,105], indicating a conserved and important role in diverse organisms. Based on CDD-PFAM analysis, *M. perniciosa *contains the largest number of thaumatins of any fungus sequenced, so far (7 thaumatins, Fig. 5A; Additional File 4).

Both PR-1 and PR-5 are induced by salicylic acid (SA) in plants [99]. Curiously, the *M. perniciosa *necrotrophic (saprotrophic) mycelia were found to produce and have tolerance to SA in axenic cultures [70]. Plants with WBD have a higher content of SA when compared with healthy plants [106]. *M. perniciosa *tolerance to SA could be explained by the expression of genes encoding salicylate hydroxylases, which were also detected in the genome (Additional File 3 – Worksheet Functional Annotation). In this scenario, high levels of SA could block the synthesis of jasmonic acid (JA), a defense compound against necrotrophic pathogens that acts as a necrosis inducer, thus rendering the plant susceptible to the spread of *M. perniciosa *[78,106]. In addition, it is possible that SA and SA-induced proteins (i.e., PR-1, PR-5) may act to limit competition from other microbial competitors during WBD progression, which would be an important component of the *M. perniciosa *pathogenicity strategy.

### Colonization: Plant cell wall degrading enzymes (PCWDE)

Degradation of hemicellulose, cellulose, pectin and depolymerization of lignin are some of the mechanisms that necrotrophic fungi use to colonize plant tissues [107]. We identified genes encoding enzymes involved in degradation of hemicellulose and cellulose, including β-1,4 cellulases, exocellobiohydrolases, endo-beta-1,4-xylanases and endoglucanases; genes encoding lignolytic enzymes including manganese dependent peroxidase and multicopper polyphenoloxidases (laccases); and genes encoding enzymes involved in pectin degradation, such as pectate lyases, polygalacturonases (pectinases) and pectin methylesterases (pectinesterase) (Additional File 3 – Worksheet Functional Annotation). *M. perniciosa *have an arsenal of plant cell wall degrading enzymes that is similar to that found in the hemibiotrophic pathogens *F. graminearum *and *M. grisea *(Fig. 5B; Additional File 9). Biotrophic *U. maydis *and symbiont *L. bicolor *have a minimal set of PCWDEs what is in accordance with their lifestyle (Fig. 5B; Additional File 9).

**Figure 7:**
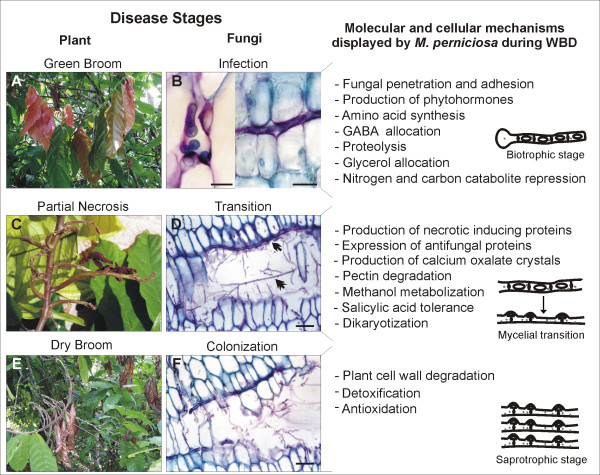
**An illustrated hypothetical model for WBD.** Model correlating classical symptoms of green and dry broom in the field (A, C and E), *M. perniciosa *development inside cacao (B: biotrophic stage, D: transition from biotrophic to saprotrophic stage, F: saprotrophic stage) and molecular and cellular events displayed by the fungus in each developmental stages, based on genes annotated in genome survey (right side of the panel). Notice in B (right side) the presence of biotrophic mycelia in the apoplast surrounded by intact living cells. Also notice in C the presence of biotrophic mycelia (arrowhead) and saprotrophic mycelia (double arrowhead) inside a necrotic region. Micrographs scales: B left side: 15 μM; B right side: 25 μM; D: 50 μM; F: 50 μM.

Pectate lyases (PL1 and PL3 according to CAZy nomenclature – ) cleave pectin, an essential component of plant cell walls. Among the fungi analyzed, *F. graminearum *and *M. perniciosa *contain the largest number of PLs (Fig. 5B; Additional File 9). Unlike *M. grisea*, the other hemibiotrophic analyzed, *F. graminearum *and *M. perniciosa *does not have specialized structures (appressoria) for non-enzymatic penetration of plants, and colonizing the apoplast by breaching the middle lamella barrier (Fig. 7). In addition, both are able to infect dicotyledons that contain cell walls with larger amounts of pectin than monocots [108]. This analysis suggests that PLs have an important role for pathogens that colonize the apoplast of dicotyledons.

### Insights into *M. perniciosa *intermediary metabolism and WBD development

Pathogen energy status and the acquisition of host metabolic compounds by the pathogen are factors that determine the outcome of disease onset. Therefore, enzymes of *M. perniciosa *involved in intermediary metabolism (i.e., reactions concerned with storing and generating metabolic energy) may have an important role during WBD (Additional File 3 – Worksheet Functional Annotation). During the first stages of WBD, *M. perniciosa *may encounter a nutrient-poor and extreme oxidative environment containing host defense molecules, such as nitric oxide (NO), an inhibitor of the cytochrome respiratory pathway [109]. Under these conditions, the activity of a NO tolerant alternative oxidase (AOX) may constitute a critical bypass mechanism for the cytochrome pathway [110-112]. A single copy of *AOX *was detected in *M. perniciosa*, and preliminary experiments indicate that this gene has a higher expression in fungi grown in nutrient-poor media (data not shown).

Following this scenario, in the low-energy status represented by the biotrophic phase, AOX may provide NAD^+ ^for the turnover of the mitochondrial tricarboxylic acid cycle (TCA) and the peroxisomal glyoxylate cycle (GLOX). Many reports have documented the importance of GLOX for phytopathogens during host infection [113-115]. All genes coding for proteins of this enzymatic pathway are present in the genomeof *M. perniciosa*, including the key enzymes isocitrate lyase and malate synthase (Additional File 3 – Worksheet Functional Annotation).

Curiously, *M. perniciosa *is able to use methanol as the only carbon source (data not shown), indicating that this fungus may have a methylotrophic metabolism. Methylotrophism have been extensively studied in methylotrophic yeasts such as *Pichia angusta *and recently methanol oxidases (MOX) have been described in filamentous fungi such as *C. fulvum *[116] and wood-degrading basidiomycete *Gloephyllum trabeum *[117]. A gene encoding a MOX was identified in *M. perniciosa *genome (Additional File 3 – Worksheet Functional Annotation). Interestingly, this gene was previously detected as being overexpressed in biotrophic-mycelia [20]. The methanol catabolism enzymes formaldehyde dehydrogenase and formate dehydrogenase (Additional File 3 – Worksheet Functional Annotation) were also found in the genome, which provides evidence that *M. perniciosa *indeed hydrolyzes methanol. Methanol is, possibly, derived from the first step of pectin hydrolyzation performed by the cell wall degrading enzyme pectin methylesterase [118], or from demethylation of lignin that occurs after manganese peroxidase and/or laccase action [117]. We can not discount the possibility that the biotrophic fungi can use the methanol produced by pectin metabolism during normal cell wall synthesis in early stages of plant expansion [119]. The possibility that *M. perniciosa *is methylotrophic resembles the methylobacteria nutritional strategy, which provides an ecological advantage over non-methylotrophic microorganisms present in the phyllosphere [120].

Previous data indicated that the utilization of glycerol, instead of fermentable sugars (i.e., glucose), is an important environmental clue for the maintenance of the biotrophic stage [18,121]. Accordingly, our group detected higher amounts of glycerol during the biotrophic fungal phase of the green brooms development than in dry brooms [13]. Additionally, *in vitro *assays showed that the shift from glucose to glycerol media increased anti-oxidative defenses of *M. perniciosa *mycelia [122]. This result correlates well with green broom environment (high content of glycerol and ROS) raising the possibility that glycerol is a critical metabolite during the initial stages of the disease cycle. Genes involved in glycerol metabolism and uptake are present in *M. perniciosa*, including a biotrophic induced aquaglyceroporin transporter [20] (Additional File 3 – Worksheet Functional Annotation), suggesting that this fungus is able to acquire extracellular glycerol.

After 35 days of WBD, glucose levels increase again, concomitant with a reduction in starch levels [13]. We found a gene model similar to a secreted glucoamylase in the *M. perniciosa *genome (Additional File 3 – Worksheet Functional Annotation). Gibberellin is an inducer of α-amylase production [123]. We can envisage a disease scenario in which the fungus produces an extracellular amylase and hormones (i.e., gibberellin) that triggers plant amylolytic activity, which then decrease starch content. The resulting glucose can be utilized by the plant since at this stage the green brooms/infected tissues are rapidly growing or even be acquired by the pathogen at the transition phase between biotrophic and necrotrophic stages.

Nitrogen starvation also appears to be a factor that influences the biotrophic lifestyle [124] and is associated with the expression of pathogenicity genes and PCWDE, mainly in hemibiotrophic and biotrophic fungi that deal with nutrient deprivation during early infection [125]. Our data from microarray and EST analysis suggests that nitrogen catabolite repression (NCR) occurs in *M. perniciosa* by the induction of GABA permease, tRNA synthetates and AROM protein [20]. As reported previously [20]*M. perniciosa *contains a gene homologous to *CLNR1 *from the hemibiotrophic fungus *C. lindemuthianum *(Additional File 3 – Worksheet Functional Annotation), a global nitrogen regulator that belongs to the AREA/NIT2 family. *CLNR1 *activates enzymes and transporters that enable uptake and catabolism of secondary nitrogen sources [126]. The depletion of *CLNR1 *impaired the fungal switch to necrotrophy [124], emphasizing the importance of nitrogen catabolism in hemibiotrophic development. Our genomic data mining identified a gene similar to *NPR2*, which encodes a regulatory protein that may act upstream of the AREA/NIT2 protein (Additional File 3 – Worksheet Functional Annotation). *NPR2 *is required for the expression of the *M. grisea *pathogenicity gene *MPG1 *[127]. In addition, genes that encode enzymes involved in alternative nitrogen sources uptake (i.e., GABA transporter, urea permease and nitrate transporter) and metabolism (i.e., urease, nitrate reductase, nitrite reductase, arginase and uricase) were identified (Additional File 3 – Worksheet Functional Annotation). The presence of such genes in *M. perniciosa *genome indicates that this fungus could allocate and utilize alternative nitrogen sources in the absence of preferential nitrogen sources (glutamine and ammonia) reinforcing our hypothesis that *M. perniciosa *suffers NCR during early stages of WBD.

## Conclusion

Our analysis of the *M. perniciosa *genome survey yielded interesting insights and clues into the molecular mechanisms underlying WBD. As far as we know, this is the first phytopathogen included in the order Agaricales sequenced. Therefore, our results support the investigation of pathogenicity mechanisms among Agaricales and Basidiomycete. In addition, we provide an approach for normalization of gene family data in a genome survey that can aid the genomics community interested in functional analysis in incomplete genome data.

Based on annotated fungal genes from this report and from previous other studies, we designed a hypothetical model for WBD that correlates plant phenotype changes that happen during the disease with the developmental progression of *M. perniciosa *(Fig. 7).

After penetration and adhesion to the plant, the biotrophic fungus slowly grows inside the apoplast causing a series of phenotype changes in cacao, such as hypertrophy and hyperplasia, phototropism and epinasty, by secreting phytohormones that unbalance cacao metabolism (Fig 7A and 7B). In addition, *M. perniciosa *has to deal with nitrogen deprivation in the apoplast, which signals the production of proteins related to the acquisition of alternative nutrient sources, proteolysis and amino acid synthesis. Furthermore, the fungus takes advantage of the increasing content of glycerol in the green broom, an important cue for biotrophic stage maintenance, by expressing aquaglyceroporins. As previously discussed [13], the starch accumulated in early stages of green brooms seems to be metabolized to glucose, which suggests an amylolytic activity exerted by fungi and/or by plant amylases. This increase of glucose is not accompanied by an increase of fructose, but by a sucrose augmentation. Since photosynthesis is not increased during WBD, we suggest that sucrose is translocated from other tissues to the green brooms. Therefore, we hypothesize that these mechanisms cause a source-to-sink transition in stem, turning green brooms into a drain of nutrients.

After numerous physiological and biochemical changes in the plant, which may be caused by the fungal infection, there occurs a transition from the biotrophic to the saprotrophic lifestyle (Fig. 7C and 7D). This change could result from the increase of nutrients in the fungal environment and may be controlled by an AREA/NIT2-like regulator. During this transition phase, the plant displays the beginning of necrosis at the distal portion of the leaves that could be due to the action of NEPs and cerato-platanins that are expressed in the biotrophic hyphae. In addition, *M. perniciosa *produces PCWDEs, such as pectinases, whose action aids the fungus in breaching the middle lamella barrier. Pectin degradation releases methanol, which in turn could be used by *M. perniciosa *as a carbon source, through the action of a MOX and other methanol metabolizing enzymes. Moreover, the calcium released from pectin disruption could be scavenged by the oxalate synthesized by the fungus, a compound that triggers cell death. The release of cell content during necrosis, and the probable aforementioned source-to-sink transition, may influence, or even be indispensable to the *M. perniciosa *switch from biotrophism to saprotrophism. Thus, the postulated carbon and nitrogen catabolite repression displayed by the biotrophic mycelia would be switched off, thus causing the mycelial change to its invasive dikaryotic/saprotrophic stage.

During disease progression, and mainly during colonization of saprotrophic hyphae, *M. perniciosa *must deal with an intense oxidative environment. Based on our analysis this stress can be overcome by the action of several anti-oxidative and detoxifying enzymes. Furthermore, we believe that *M. perniciosa *exerts a negative control on plant defense against necrotrophic/saprotrophic fungi by producing salicylic acid, which would limit competition by other fungi by the action of antifungal proteins. Finally, after alternating wet and dry periods, the formation of the basidiomes produced by saprotrophic hyphae occurs.

Latin American cacao crops suffer tremendous damages caused by WBD, which mainly affects small acreage farmers. Such impact in the relatively primitive cacao cultivation system not only affects the socio-economic status of farmers but also the preservation of the rainforest. The demand for strategies that limit cacao diseases requires an intense effort in understanding the pathogenicity and plant resistance mechanisms. Further sequencing projects of cacao and its pathogens will serve as a background for the integration of transcriptomics, proteomics and metabolomics of these species in a systems biology approach. Such initiatives will provide tools for biological control, crop management and cacao biotechnology to combat cacao diseases. We believe that our report is the first step towards such an integrative initiative and provides insights into the molecular mechanisms of WBD which can aid the cacao's WBD-concerned community to develop control strategies for this plant-fungus interaction.

## Methods

### Biological material, libraries construction and sequencing

Total DNA was extracted from saprotrophic hyphae of *M. perniciosa *strain FA553 (CP02) maintained in Malt Yeast Extract Agar (Difco) at 27°C. DNA was extracted from grounded mycelia by incubation in CTAB buffer (CTAB 3%, NaCl 1.4 M, EDTA 20 mM pH 8.0, Tris-HCl 10 mM pH 8.0, PVP 1.0%, β-mercaptoethanol 0.2%) at 65°C during 30 min; followed by one phenol:chloroform:isoamyl alcohol (25/24/1) wash, precipitation with sodium acetate pH 5.2 (0.1 Vol) and cold 100% ethanol (2 Vol). DNA was eluted in deionized water and sheared by nebulization and sonication into fragments of approximately 2 Kbp, which were size selected on agarose gels and purified with S.N.A.P. Gel Purification Kit (Invitrogen – Life technologies, USA). DNA fragments were blunt-end ligated into the pCR4Blunt plasmid (Invitrogen – Life technologies, USA). Approximately 50 genomic libraries were constructed, each one corresponding to individually growing cultures. Sequencing was done in an ABI Prism 3700 sequencer (Applied Biosystems, USA).

### Clustering

The resulting chromatograms were submitted to the *M. perniciosa *database and subjected to automatic base calling using the software PHRED [128]. The contaminating vector sequences and low quality shotgun reads, without at least 100 bp with phred note ≥ 16 were trimmed by using the program LUCY [129]. Shotgun reads showing significant sequence similarity (BLASTn, E-value ≥ 1E-30) with *M. perniciosa *mitochondrial sequences (see above) were removed from subsequent assemblies. The clustering and assembly were performed using the software PHRAP . Afterwards, low quality regions of singlets previously evaluated by LUCY (the last window of 10 bp that has an average probability of error given by phred ≤ 10) were trimmed. The remaining clusters were subjected to similarity searches against the NCBI non-redundant protein and nucleotide database using the BLASTx and tBLASTx, respectively, with an E-value cutoff of 1E-5.

### Genome Length Statistical Validations

Statistical analyses of the genome length were performed using two approaches. The first one was based on the Dog genome survey using counting of start positions offsets for overlapping reads [31] (for further details see Additional File 1). The second was based on Lander Waterman Theory, [34] and their applications [35,36], which estimate the theoretical values of expected number of clusters (contigs + singlets), contigs, gaps, average cluster size and average gap size, using the effective average read length (L), the total number of reads in the assembly (N) and the estimated genome size (G). L is the average number of base pairs of a read that contributes to the contig through parsing of ace file . In our analysis L was equal to 550 bp. In order to estimate the gap size distribution in the *M. perniciosa *genome survey, we performed a comparison between a set of eukaryotic core protein (generated by CEGMA pipeline [37]) and *M. perniciosa *contigs using TBLASTN with threshold of 1e-10 for the E-value (Further details in Additional File 1). The estimation of misassembled sequences due to repetitive regions in the genome was performed using the integrated pipeline amosvalidate [38] (Further details in Additional File 1).

### Gene Finding

#### EST against genome alignments

The alignment of ESTs with genomic sequences was performed using the package GeneSeqer [130] with the pre-built *Aspergillus *intron model. 300 highly confident introns were selected and used as an input for Exalin program [39] that is able to build a splice site model for an organism. The positions of the splice sites as assigned by Exalin were used to rank overlapping gene predictions (see below).

#### *Ab initio *gene models prediction

The *ab initio *gene models prediction was performed with the trainable, open source gene predictors AUGUSTUS [40], SNAP [41] and GENEZILLA [42]. Ten copies of an artificial sequence of 240 Kbp (total of 2.4 Mbp) formed by the concatenation of the *M. perniciosa *ESTs coding regions, together with a *C. cinerea *gene dataset containing 1.2 Mbp were submitted to "pre-training" in AUGUSTUS gene predictor. *M. perniciosa *resulting predictions were compared with the protein databank NR using BLASTp. The predictions with similarities in NR, and with coverage ≥ 90%, were selected. After redundancy elimination, *M. perniciosa *gene models were used to train the three gene finders aforementioned. Predictions with less than 30 amino acids were eliminated, and the remaining predictions were grouped in overlapping clusters.

#### *Ab initio *gene models ranking

The predictions in each overlapping cluster were ranked according to the criteria used by the Fungal Genome Initiative at Broad institute . In each cluster, the "best" *ab initio *gene model according to the stipulated criteria was selected for functional annotation. The criteria for the ranking of the gene models were the following:

1. Manual annotation had priority over all other evidences;

2. Predictions with EST evidences had priority over the predictions without EST evidences;

3. If two predictions had EST evidences, the one with more splice sites in exact agreement with ESTs had priority;

4. Prediction with similarities with known proteins had priority. A prediction was considered to be similar to some known protein if it had an E-value of at most 1e-10 (BLASTP against NR+*Phanerochaete chrysosporium *protein set);

5. If two predictions had similarity with known proteins, the one with better coverage score had priority. The coverage score was defined as 2 × CP × CH/(CP+CH), where CP is the coverage of the prediction and CH is the coverage of the similar protein;

6. In clusters without similarity with known proteins and without EST evidence, the priority was for AUGUSTUS, SNAP and GENEZILLA, in this order. This criterion was chosen according to the performance of the three programs in a dataset of 60 genes structures visually inspected.

A final filter discarded gene predictions reported by only one program, without similarity to known proteins and without EST evidence.

#### Extrinsic gene models prediction

The extrinsic gene model predictions were performed by two methodologies. First, 17,991 contigs and 7,065 singlets were submitted to similarity analysis in a databank containing BLASTX-NR plus *Phanerochaete chrysosporium *proteins. The genomic regions containing homologues in this databank were selected and assigned as putative gene models. GenomeThreader [131] program was used to make protein-DNA spliced alignments between the BLAST first hit against and the genomic sequence, serving as a guide to delimit the start and stop codons and exon-intron boundaries of the regions of the contigs containing similarity with GenBank.

Concurrently, *M. perniciosa *ESTs aligned with genomic clusters (see above) were inspected to verify if the region in which they aligned contained a BLAST extrinsic prediction gene model. These extrinsic gene models (EST and BLAST) were compared with each other to evaluate the amount of gene models predicted by these methods. After these comparisons, the extrinsic gene models were divided into 4 datasets:

(i) ESTMODELS: retrieved from the spliced alignments of the ESTs against the genomic clusters not covered to a BLAST extrinsic gene models prediction. Low score spliced alignments and ESTs that seen clearly to be UTR of a neighboring prediction were not included.

(ii) BLASTMODELS: derived from BLAST extrinsic gene models predictions analysis covering genomic regions without EST evidence.

(iii) COMBINEDMODELS: gene models derived from genomic sequence regions with BLAST hit and EST evidence.

(iv) CURATEDMODELS: extrinsic predictions manually annotated for manual correction of merged or split predictions.  The genomic survey and gene models nomenclature are depicted in additional file 10.

#### tRNA prediction

For tRNA prediction, the tRNAscan-SE program [132] was taken into account with the default parameters, which searched for conserved sequences and the characteristic secondary structure of tRNAs.

#### MCL-families clustering

MCL graph clustering algorithm was applied to generate *M. perniciosa *gene families using WU-TBLASTx "all against all" as the tool used for aligning the gene models [43].

#### Normalization of gene family data

The normalization of gene family data was performed by comparison between *C. cinerea *proteins and *M. perniciosa *gene models, using TBLASTN with 1E-10 of E-value threshold. The number of gene models similar to a *C. cinerea *protein was plotted according to protein length. The equation that estimates the number of gene models representing the same protein was generated using linear regression fitting. This equation was used to estimate a normalization factor to each MCL family and CDD-PFAM domain according to the average of protein length of their members.

### Automatic Annotation and Metabolic Maps

The automatic annotation program AutoFACT [47] was used for functional annotation of gene models. The set of coding sequences from gene models were submitted to similarity searches against the UNIREF100, UNIREF90, NR, and KEGG databases using BLASTx (E-value ≤ 1E-5) and against CDD-PFAM using RPS-BLAST (E-value ≤ 1E-5) [48]. These results were submitted to AutoFACT, which searches for a consensus in the results and output descriptions and statistics about protein domains and families.

For an inference of *M. perniciosa *metabolic maps, we used Pathway Tools (version 11.0), a software of BioCyc databases [49], which generates a metabolic map from a previously annotated genome. The pathways that are probably present in the genome are imported from a reference database, following the Pathway Tools parameters [133]. The annotated genome input was obtained from EST manual annotation and from *M. perniciosa *gene models AutoFACT annotation, using as main information the product name and, if available, E.C. numbers. Metabolic pathways of interest were manually annotated for the elimination of false positives.

The analysis of transporters was made based on a BLASTX search of *M. perniciosa *gene models and other fungal genes against TCDB (Transport Classification Database – ), using a threshold of E-value 1E-05. All classes from third level that contained at least one species with 2% or more of representations were separately represented in the results.

### Selection and expression confirmation of no hits *ab initio *gene models

The selection of *ab initio *gene models was performed using a SQL query wizard. As input, we ask for gene models without similarity in GenBank NR, which encoded proteins that contained at least 2 cysteines and a signal peptide, previously identified by Signal-P 3.0 program [134]. 74 gene models were then selected and their nucleotide sequences were used as template for the design of primers nested in: (i) the sequence encoding the putative signal peptide (SPE); (ii) the sequence encoding the putative first amino acid of mature protein (MAT) and; (iii) the sequence containing the putative stop codon (END). The latter was designed in reverse complement ("reverse") to allow gene amplification using the other two primers ("forward"). RT-PCR analysis was performed to validate the expression of *ab initio *predicted gene models. RNA from saprotrophic mycelia was extracted using hot-phenol method with modifications [135]. Equal amounts of total RNA from CP02 saprotrophic mycelia cultures (24 h, 48 h, 4 days and 7 days) were mixed. After DNase (Invitrogen, USA) treatment, 2 μg of total RNA was reverse transcribed using Superscript RTII (Invitrogen, USA) in a total volume of 20 μL, following the manufacturer's instructions. PCR reactions were conducted according to primers (MWG, Imprint Genetics Corp) temperature of melting (TMs).

## Abbreviations

ABC: ATP Binding Cassette; AOX: Alternative oxidase; bp: base pairs; COC: calcium oxalate crystals; CTAB: Cetyl trimethylammonium bromide; EDTA: Ethylenediaminetetraacetic acid EST: Expressed Sequence Tag; FPR: Frosty Pod Rot; GA: Gibberellin; GABA: Gamma-aminobutyric acid; GGPP: Geranylgeranyl Diphosphate; IAA: indole-3-acetic acid; JA: Jasmonic acid; Kbp: One thousand base pairs; LW: Lander Waterman; Mbp: One million base pairs; MOX: Methanol oxidase; NAD(P): Nicotinamide adenine dinucleotide phosphate; NCR: nitrogen catabolite repression; NEP: Necrosis and ethylene-inducing proteins; NO: Nitric oxide; PCD: Programmed cell death; PCWDE: Plant cell wall degrading enzymes; PVP: Poly(vinylpyrrolidone); ROS: Reactive oxygen species; RT-PCR: Reverse Transcription-Polymerase Chain Reaction; SA: Salicylic acid; PCR: Polymerase Chain Reaction; PR: Pathogenesis related protein; TCA: Tricarboxylic acid cycle; TE: Transposable Elements; TM: temperature of melting; tRNA: transfer RNA; UTR: untranslated region; WBD: Witches' Broom Disease.

## Authors' contributions

JMCM conceived and wrote the article, was responsible for gene models annotation, gene density analysis, primer design and data interpretation. MFC conceived bioinformatics analysis and was responsible for genome assembly, BLASTs, statistical analysis and data interpretation. GGLC was responsible for gene models predictions, Genome Threader analysis, MCL clustering analysis and gene browser design and implementation. EFF participated in genome assembly, was responsible for transporters analysis and annotation, and made figures edition. LPP was responsible for AutoFACT and BioCyc implementation and analysis. JR was responsible for EST library construction and sequencing and participated in genome annotation. CC, DMC, AFC and HC were responsible for *M. perniciosa *DNA extraction, genome shotgun, cloning and maintenance of genomic libraries. ROV was responsible for gene browser design and implementation, tRNA analysis and *M. perniciosa *sketches. RCE performed RT-PCR and aided in primer design. OG, DPTT BVO and MHM participated in genome annotation and were responsible for the characterization of genes encoding NEPs, AOX, MOX and auxin biosynthetic proteins, respectively. ABLP was responsible for EST library construction. MCSR was responsible for microscopic analysis. MRAR participated in genome assembly and in the development of bioinformatics tools. LABC, KPG, MSG, AGN and LBB were coordinators of sequencing groups. JPMN was responsible for sequencing and libraries maintenance. MJG and BAB participated in final manuscript elaboration and provided additional sequencing in order to close gene gaps. LWM participated in pathogenicity gene data interpretation and final manuscript elaboration. JCC was the co-coordinator of *M. perniciosa *genome project and coordinated UESC sequencing group. GAGP conceived and was the coordinator of *M. perniciosa *genome project, participated in data interpretation and in final manuscript elaboration.

## Supplementary Material

Additional file 1 **Genome statistical validations.** A) Estimation of genome length using dog genome survey protocol, B) Estimate of distribution of gap sizes in *M. perniciosa *genome assembly, C) Estimate of misassembly sequences due to repetitive regions. Click here for file

Additional file 2 **MCL analysis of *M. perniciosa *gene models.** All predicted proteins were compared all-against-all using WU-TBLASTX. A score (-log (E-value)) for each pair of proteins (u, v) with significant BLAST hits (Evalue ≤ 1e-5) was assigned. The MCL algorithm (inflation parameter 2.0) was applied to find clusters in this graph. This method is fully automatic and protein clusters reported were not subjected to manual curation. ID: number of the MCL family; #members: number of gene models present in each family; Norm factor: factor used to normalize the number of gene models present in each family (see methods); Norm#members: normalized number of gene models present in each family. Annotation: words associated to each family after correlation of gene models with AutoFACT annotation. In parenthesis are the occurrence numbers of each word. Each worksheet shows the ranking of families using normalization factor (Rank_Norm) or not using this factor (Rank_Non_Norm). Click here for file

Additional file 3 **Functional annotation of *M. perniciosa *gene models discussed in this paper.** ID: gene model; First Hit (BLASTX-NR): Most similar sequence in GenBank; E-value: E-value of most similar sequence; AutoFACT annotation: automatic annotation by AutoFACT; AutoFACT E-value: E-value of AutoFACT annotation; EST: presence (Y) or absence of an EST aligned in this gene model; MCL family: family annotated by MCL analysis. Worksheet P450: annotation of gene models similar to cytochrome P450 monooxygenases; Worksheet transposons: classification and annotation of gene models similar to transposable elements; Worksheet unknown gene families; annotation of top 20 MCL unknown gene families; Worksheet functional annotation: classification and annotation of gene models similar to efflux transporters, anti-oxidative enzymes, phytohormones biosynthesis related proteins, pheromone receptors, salicylate hydroxylases, effectors/elicitors/pathoghenicity associated proteins, cell wall degrading enzymes and intermediary metabolism enzymes (cytochrome pathway bypass, Glyoxylate pathway and oxalate formation, glycerol uptake and metabolism, extracellular sugar degrading enzymes and nitrogen regulation, uptake and metabolism enzymes), EC = enzyme classification ; Worksheet transporters; Relative percentage of transporters distribution in fungi genomes (see methods). Click here for file

Additional file 4 **Ranking of CDD-PFAM families annotated in *M. perniciosa*.** Gene models were annotated based on CDD-PFAM-ID and ranked. This analysis was performed with other fungi genomes, which CDD-PFAM entries were classified according to *M. perniciosa* ranking. CDD-ID: CDD entry; PFAM Domain: PFAM entry; #Hits Domains: number of gene models containing each CDD-PFAM domain; %Hits Domains: percentage of gene models containing each CDD-PFAM domain in relation to total number of gene models containing a CDD-PFAM domain; %Hits PTN: Percentage of gene models containing each CDD-PFAM domain in relation to total number of M. perniciosa gene models; Rank: non-normalized *M. perniciosa* CDD-PFAM ranking; PTNS: proteins in each organism; Norm Factor: factor used to normalize the number of gene models containing each CDD-PFAM domain; # Hits Domains Norm: normalized number of gene models containing a CDD-PFAM domain; %Hits Domains Norm: percentage of gene models containing each CDD-PFAM domain in relation to total number of gene models containing each CDD-PFAM domain; %Hits PTN Norm: Normalized percentage of gene models containing each CDD-PFAM domain in relation to total number of *M. perniciosa* gene models; Rank Norm: normalized *M. perniciosa* CDD-PFAM ranking. Worksheets show the ranking of CDD-PFAM domains using normalization (Rank_Norm) or not using normalization (Rank_Non_Norm). Click here for file

Additional file 5 **BioCyc comparison between *S. cerevisiae *and *M. perniciosa *metabolic pathways.** Worksheet Compounds: Comparison of number of reactions in each organism containing the compounds described in the table; Worksheet pathways: Comparison of number of pathways in each organism present in each pathway class. The two largest top-level classes, Biosynthesis and Degradation/Utilization/Assimilation, are broken down further to show the distribution of pathways among their next-level subclasses. Click here for file

Additional file 6 **Annotation of gene models with no similarity in BLASTX-NR encoding hypothetical small secreted proteins containing at least 2 cysteines.** ID: gene model; # residues: number of amino acids of predicted protein encoded by the gene model; # cysteines: number of cysteines in predicted protein; Binomial RT; statistical analysis of cysteines presence in gene models (see methodological details in the file). Click here for file

Additional file 7 **Primers used in the amplification of no hits gene models encoding hypothetical small secreted proteins containing at least 2 cysteines.** ID: gene model; Set of primers: Group of primers used for the amplication of a gene model. Primer sequence: sequence of primers (SPE – nested in sequence encoding the putative signal peptide; MAT – nested in sequence encoding the putative first amino acid of mature protein; END – nested in sequence containing the putative stop codon). Amplification: positive (Y) or negative (N); EST: presence (Y) or absence (N) of an EST aligned in this gene model; Length: length of amplicon in genomic and cDNA using two combinations of primers (SPE-END; MAT-END). Click here for file

Additional file 8 **Examples of amplifications of no hits gene models.** PCR amplicons were run on 1% agarose gels. SPE: amplicons resulted from amplification with SPE and END primers; MAT: amplicons resulted from amplification with MAT and END primers; Ctl: water as template (control); Gen: genomic DNA as template; Glu: cDNA from saprotrophic mycelia grown in glucose as template; Cac: cDNA from saprotrophic mycelia grown in cacao extract as template; M: DNA molecular marker. Click here for file

Additional file 9 **Comparison of plant cell wall degrading enzymes in fungi that interact with plants.** PFAM entries were correlated with the CAZy nomenclature  of plant cell wall degrading enzymes. Click here for file

Additional file 10 **Genomic survey sequences and gene models nomenclature.** Click here for file
