# Supplementary material for: A genome survey of Moniliophthora perniciosa gives new insights into Witches' Broom Disease of cacao
Source: BMC Genomics. 2008 Nov 18;9:548. doi: 10.1186/1471-2164-9-548 (PMC2644716; doi:10.1186/1471-2164-9-548)
Supplement: Additional file 1 — Genome statistical validations. A) Estimation of genome length using dog genome survey protocol, B) Estimate of distribution of gap sizes in M. perniciosa genome assembly, C) Estimate of misassembly sequences due to repetitive regions. [file 1471-2164-9-548-S1.pdf]

Additional File 1: Genome Statistical Validations

A) Estimation of genome length using dog genome survey protocol.

From samples of reads (20,000 or 50,000), the numbers and positions of overlaps that began 3 or more bases downstream from the 5' end of the read were computed. In order to eliminate reads from repetitive regions, only reads with fewer than 3 overlaps beginning in this region were considered. For a given window in this region of 100 bases (***L***), the number of overlaps (***O***) beginning in that window is tabulated for each read:

	20,000 reads		50,000 reads	
# Overlaps (***O***)	# reads	% reads (***F*** = frequency)	# reads	% reads (***F*** = frequency)
0	14,657	79,9% (0.799)	34,233	79,5% (0.795)
1	2,946	16,1% (0.161)	7,1	16,5% (0.165)
2	751	4,1% (0.041)	1,71	4,0% (0.04)
Total	18354	100%	43043	100%

This results in a Poisson distribution and, for 50,000 reads, the average distribution (λ), meaning the mean value of overlaps, gives:

$$\lambda = 0 \,.\, (0.795) + 1 \,.\, 0.165 + 2 \,.\, 0.04 = 0.245$$

The same value is obtained using 20,000 reads , which shows that these samples of reads are statistically significant.

Another possibility to obtain λ is estimating the probability (***p***) of a read beginning in a window length (***L***) of 100 bp. This probability is p=L/G, where ***G*** is the genome length. Equating λ to ***n*** *x* ***p***, where ***n*** is the total number of reads used in the assembly (94,611), we have:

$$\lambda = \boldsymbol{n \times L/G}$$

$$\boldsymbol{G = n \times L/\lambda}$$

$$\boldsymbol{G = 94{,}611 \times 100/0.245}$$

$$\boldsymbol{G = 38.7\ \mathrm{Mb}}$$

B) Estimate of distribution of gap sizes in *M. perniciosa* genome assembly.

In order to obtain more information about gaps size distribution, we performed a comparison between a set of eukaryotic core proteins (CEGMA pipeline [Parra *et al.,* 2007]) and *M. perniciosa* contigs, using TBLASTN with threshold of E-value 1e-10.

Since these proteins are supposed to exist in all eukaryotic organisms, we assumed that *M. perniciosa* should have at least one copy from each protein. Then, if a protein from this set was not found in *M. perniciosa* genome, it should be exclusively because of the gaps in the genome draft. The figure below shows the distribution of protein length in function of number of proteins used in this analysis. The analysis clearly indicates that predominantly eukaryotic core proteins that are “no hits” in *M. perniciosa* genome are small ones. For instance, 80% of “no hits” proteins have size smaller than 300 aa, and the “no hits” average protein size is around 167 aa. We believe that this result is indirectly related with the distribution of gaps length. Therefore, we assume that the average gap length is around 500 bp (3 x 167 aa). The standard deviation of this distribution was ± 100 aa (± 300 bp).

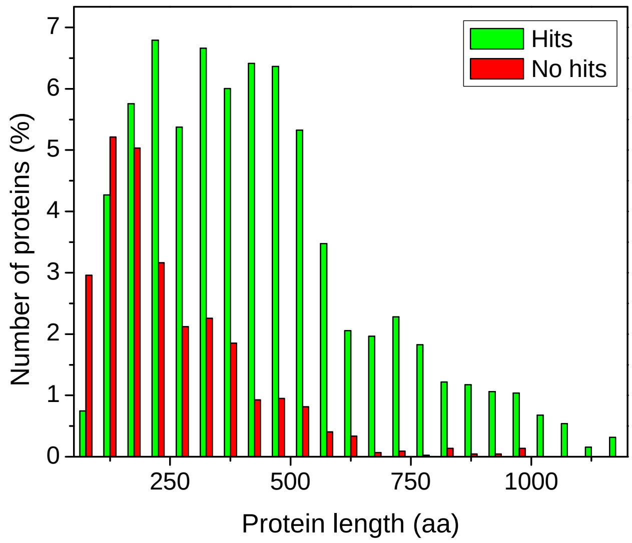

Figure Additional file 1: Correlation between the number (axis y) and the percentage (axis x) of eukaryotic core proteins selected by CEGMA that have similarity with *M. perniciosa* genome survey sequences.

C) Estimate of misassembly sequences due to repetitive regions

In order to estimate the number of sequences misassembled because of repeat regions we used the integrated pipeline for assembly validation, called amosvalidate [Philippy *et al*., 2008]. This program is able to identify regions into contigs that are over-represented in a set of reads. Using the length of these regions (***m***) and the information about how many times these regions are covered (***c***) in relation to the coverage of non-repeat regions (***C***), it is possible to estimate the number of repeat sequences in the genome. We detected 664 contigs containing repeat regions totalizing 1.1 Mb.

$\sum (\boldsymbol{m}) = 1.1$ Mb

To obtain the number of Mb collapsed in this 1.1 Mb, we calculated:

$\sum(\boldsymbol{m} \times \boldsymbol{C}/\boldsymbol{c}) = 6.3$ Mb

Therefore, the total number of base pairs in repeat regions is 7.4 Mb.

References:

Parra G, Bradnam, K, Korf, I. **CEGMA: a pipeline to accurately annotate core genes in eukaryotic genomes**, *Bioinformatics*. 2007, **23** (9) 1061-1067.

Phillippy AM, Schatz MC, Pop M., **Genome assembly forensics: finding the elusive mis-assembly,** *Genome Biol*. 2008, **9**(3):R55.
