# Supplementary figures and images for: A genome survey of Moniliophthora perniciosa gives new insights into Witches' Broom Disease of cacao
Source: BMC Genomics. 2008 Nov 18;9:548. doi: 10.1186/1471-2164-9-548 (PMC2644716; doi:10.1186/1471-2164-9-548)

**Additional File 8**

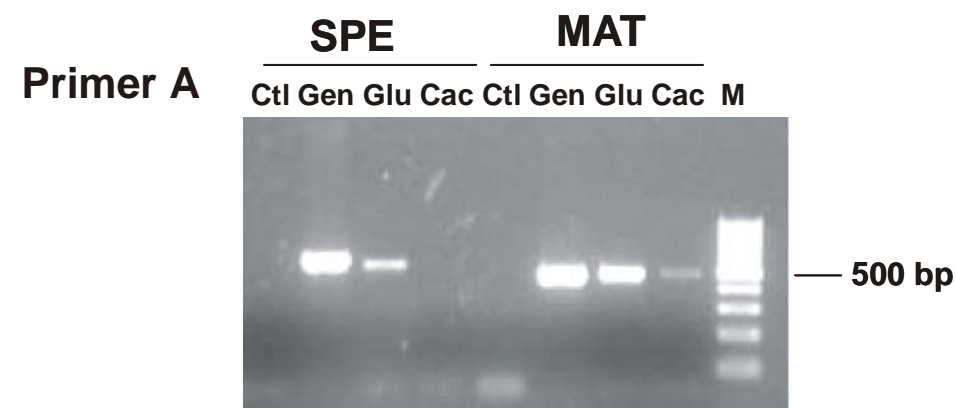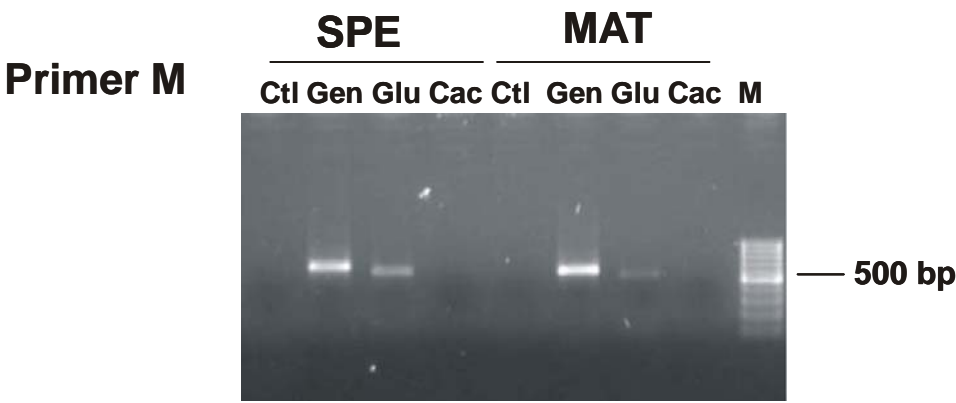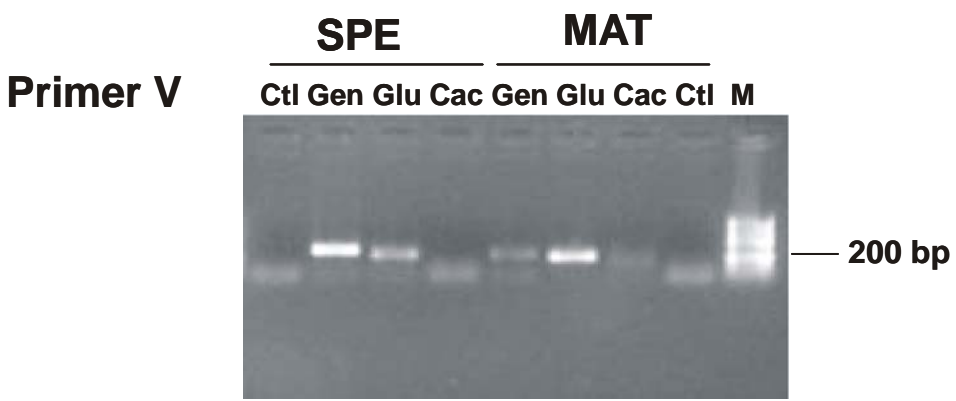

Supplement: Additional file 8 — Examples of amplifications of no hits gene models. PCR amplicons were run on 1% agarose gels. SPE: amplicons resulted from amplification with SPE and END primers; MAT: amplicons resulted from amplification with MAT and END primers; Ctl: water as template (control); Gen: genomic DNA as template; Glu: cDNA from saprotrophic mycelia grown in glucose as template; Cac: cDNA from saprotrophic mycelia grown in cacao extract as template; M: DNA molecular marker. [file 1471-2164-9-548-S8.pdf]
