# Supplementary material for: A genome survey of Moniliophthora perniciosa gives new insights into Witches' Broom Disease of cacao
Source: BMC Genomics. 2008 Nov 18;9:548. doi: 10.1186/1471-2164-9-548 (PMC2644716; doi:10.1186/1471-2164-9-548)
Supplement: Additional file 9 — Comparison of plant cell wall degrading enzymes in fungi that interact with plants. PFAM entries were correlated with the CAZy nomenclature of plant cell wall degrading enzymes. [file 1471-2164-9-548-S9.pdf]

## Additional File 9: Comparison of plant cell wall degrading enzymes in fungi that interact with plants.

| Pfam domain[a] | CAZY[b] | MP | MG | FG | CC | PC | NC | LB | UM |
|---|---|---|---|---|---|---|---|---|---|
| pfam00150 | GH5 | 4 | 10 | 4 | 9 | 11 | 6 | 7 | 6 |
| pfam01341 | GH6 | 3 | 3 | 1 | 0 | 1 | 3 | 0 | 0 |
| pfam00840 | GH7 | 5 | 5 | 2 | 6 | 8 | 6 | 0 | 0 |
| pfam00759 | GH9 | 2 | 0 | 0 | 1 | 1 | 2 | 1 | 1 |
| pfam00331 | GH10 | 8 | 7 | 5 | 6 | 6 | 4 | 0 | 2 |
| pfam00457 | GH11 | 2 | 5 | 2 | 6 | 1 | 2 | 0 | 1 |
| pfam02015 | GH45 | 2 | 1 | 1 | 0 | 0 | 1 | 0 | 3 |
| pfam03443 | GH61 | 13 | 20 | 12 | 33 | 14 | 14 | 7 | 0 |
| pfam00544 | PL1 | 6 | 2 | 9 | 1 | 0 | 0 | 0 | 1 |
| pfam03211 | PL3 | 4 | 1 | 7 | 2 | 0 | 1 | 0 | 0 |
| pfam00295 | GH28 | 4 | 3 | 6 | 3 | 3 | 2 | 8 | 1 |
| pfam01095 | CE8 | 2 | 0 | 3 | 0 | 2 | 1 | 3 | 1 |

a - Number of PFAM domain
b - Nomenclature of plant cell wall degrading enzymes according to CAZy (www.cazy.org).
Mp = *Moniliophthora perniciosa*, Mg = *Magnaporthe grisea*, Fg = *Fusarium graminearum*, Cc = *Coprinopsis cinerea,* Pc = *Phanerochaete chrysosporium,* Um = *Ustilago maydis,* Lb =*Laccaria bicolor*
