# Supplementary material for: A genome survey of Moniliophthora perniciosa gives new insights into Witches' Broom Disease of cacao
Source: BMC Genomics. 2008 Nov 18;9:548. doi: 10.1186/1471-2164-9-548 (PMC2644716; doi:10.1186/1471-2164-9-548)
Supplement: Additional file 10 — Genomic survey sequences and gene models nomenclature. [file 1471-2164-9-548-S10.pdf]

## Genomic survey sequences and Gene models nomenclature

### Genomic survey sequences nomenclature

Genomic survey sequences were identified according to the system established for the Witches' Broom Genome Project, which considers the following data: organism and strain, library construction strategy, library number, plate number, position in 96-well plate, sequencing institution, and primer used for sequencing.

System: LLAA – LA – AAA – NNN – LNN – LL – L
Exemple: CP02 – EC – 001 – 001 – A01 – UC - F
1 2 3 4 5 6 7

L= Letter; N= Number; A= alpha-numeric

1. Organism refers to the species and isolate used for the study:
a. CP= *Moniliophthora perniciosa*
b. 02= isolate CP02

2. Library construction strategy refers to the genomic libraries
a. S0= genomic shotgun library cut with enzyme *Rsa*I to a fragment size of 0.5 to 2 Kb;
b. S1= genomic shotgun library cut by nebulization to a fragment size of 0.5 to 2 Kb
c. S3= genomic shotgun library cut by nebulization to a fragment size of 2 to 4 Kb

3. Library number refers to the number of independent ligation and transformation events for each library:
a. 001= first cloned library
b. 002=second cloned library, etc.

4. Plate Number refers to the 96-well plate containing individual white colonies from each transformation:
a. 001= first plate
b. 002=second plate; etc.

5. Position of clone inside the 96-well plate
a. A01= row A, column 01; etc.

6. Sequencing institution (http://www.lge.ibi.unicamp.br/vassoura):
a. UC= Universidade Estadual de Campinas (UNICAMP)
b. UE= Universidade Estadual de Santa Cruz (UESC)
c. CL= Comissão do Plano da Lavoura Cacaueira (CEPLAC)
d. EM= EMBRAPA
e. FS= Universidade Estadual de Feira de Santana

7. Primer used for sequencing:
a. F= Forward primer
b. R= Reverse primer
c. G= Resequencing of the same clone with forward primer

**Gene models nomenclature**

Gene models nomenclature followed the criteria described: Contig or singlet at which a gene model is present; *ab initio* gene finder program (AUGUSTUS, SNAP, Genezilla) or extrinsic prediction (ESTMODELS, BLASTMODELS, COMBINEDMODELS, CURATEDMODELS); position of gene model in the contig sequence.

Contig17878_BLASTMODELSv1_g2
CP02-S2-000-143-B10-UE.F_Genezilla_v6x_g1
1 2 3

1. Contig or singlet at which a gene model is present
a. Contig17878 (contig)
b. CP02-S2-000-143-B10-UE.F (singlet)

2 .*Ab initio* gene finder or extrinsic prediction
a. *Ab initio*: AUGUSTUS, SNAP, Genezilla
b. Extrinsic: ESTMODELS, BLASTMODELS, COMBINEDMODELS, CURATEDMODELS

3. Position of gene model in the contig sequence (from 5´ to 3´)
a. g1: Closer to 5´
b. g2: Farther from 5´
